# Cremastrae Pseudobulbus Pleiones Pseudobulbus (CPPP) Against Non-Small-Cell Lung Cancer: Elucidating Effective Ingredients and Mechanism of Action

**DOI:** 10.3390/ph17111515

**Published:** 2024-11-11

**Authors:** Yuxin Cao, Zhuangzhuang Hao, Mengmeng Liu, Jingwen Xue, Yuqing Wang, Yu Wang, Jiayuan Li, Yifan Lu, Chunguo Wang, Jinli Shi

**Affiliations:** 1 School of Chinese Medica Materia, Beijing University of Chinese Medicine, Beijing 100029, China; 20210941403@bucm.edu.cn (Y.C.); bucmhzz@126.com (Z.H.); lmm_plant@163.com (M.L.); xuejingwen_10@163.com (J.X.); bucmwangyg@163.com (Y.W.); wangyu19970721@163.com (Y.W.); ljy15612930585@163.com (J.L.); asd12382022@163.com (Y.L.); 2 Institute of Chinese Materia Medica, Beijing University of Chinese Medicine, Beijing 100029, China; wangcg1119@126.com

**Keywords:** Cremastrae Pseudobulbus Pleiones Pseudobulbus, non-small-cell lung cancer, effective ingredient combinations, spectrum–effect relationships, serum pharmacochemistry, action mechanisms, AMPK-mTOR-ULK1/BMF signaling pathway

## Abstract

Cremastrae Pseudobulbus Pleiones Pseudobulbus (CPPP) is derived from the dried pseudobulb of the orchid family plants *Cremastra appendiculata* (D.Don) Makino, *Pleione bulbocodioides* (Franch.) Rolfe, or *Pleione yunnanensis* Rolfe, and has the properties of clearing heat, detoxification, resolving phlegm, and dispersing nodules. It is frequently used for the treatment of various malignant tumors in clinical practice, especially lung cancer. CPPP is divided into two commercial specifications in the market, Maocigu (MCG) and Bingqiuzi (BQZ). However, owing to a lack of appropriate research strategies, the active ingredients and molecular mechanisms involved have not yet been clarified. This study intended to discover the combination of effective anti-lung-cancer ingredients in CPPP and explore their potential mechanisms of action. In this study, UHPLC-MS fingerprints of MCG and BQZ were established separately. Inhibitory effects on the proliferative viability and migratory ability of A459 and H1299 cells were evaluated as pharmacodynamic indicators. GRA and BCA were used to determine spectrum–effect relationships. Next, the identification and analysis of components of drug-containing serum were performed using UHPLC-Q-Exactive Orbitrap MS. Then, the results of the two analyses were combined to jointly screen out the anti-lung-cancer candidate active monomers of CPPP, and their in vitro activities were verified. Afterward, all effective ingredient combinations of MCG (MCGC) and BQZ (BQZC) were prepared according to their contents in the original medicinal materials. Their anti-lung-cancer activities in vitro and in vivo were compared and verified. Finally, we used the human lung cancer cell line A549 and the Lewis tumor xenograft model to investigate how BQZC would influence autophagy and apoptosis processes and the mechanisms involved. Overall, 11 predominant anti-lung-cancer active ingredients from CPPP were screened. Next, MCGC and BQZC were prepared according to their contents in the original medicinal materials, respectively, and their anti-tumor effects were equivalent to those of the original materials in vitro and in vivo. We found that BQZC could inhibit lung cancer cell growth and induce protective autophagy and apoptosis in lung cancer cells by activating the AMPK–mTOR–ULK1/BMF signaling pathway. These results provide important evidence for the clinical application and deep development of CPPP against tumors.

## 1. Introduction

Lung cancer is one of the most common malignant tumors, with an extremely high morbidity and mortality, and can be divided into small-cell lung cancer (SCLC) and non-small-cell lung cancer (NSCLC). The latter is a widespread concern due to its high incidence rate (85% of lung cancer) and low survival rate (13–60%) [1,2]. Although significant progress has been made in research on the treatment of NSCLC in recent years, the mortality rate of patients remains high [3,4]. Therefore, it is urgent to search for new highly efficient and low-toxicity drugs for NSCLC treatment. Continuous in-depth research on traditional Chinese medicine (TCM) has shown advantages in the treatment of lung cancer, such as multiple pathways, multiple targets, fewer toxic side effects, and a lower susceptibility to drug resistance [5].

Cremastrae Pseudobulbus Pleiones Pseudobulbus (CPPP) is derived from the dried pseudobulb of *Cremastra appendiculata* (D.Don) Makino, *Pleione bulbocodioides* (Franch.) Rolfe, or *Pleione yunnanensis* Rolfe, among which the former is commonly known as “Maocigu” (MCG), while the latter two are commonly known as “Bingqiuzi” (BQZ). The market is mainly divided into these two commercial specifications, which significantly differ in their appearance characteristics and chemical composition [6]. However, there are no reports on whether their medicinal effects are the same. CPPP is cool in nature and slightly pungent and sweet in taste. It also has the effects of clearing heat, detoxification, resolving phlegm, and dispersing nodules [7,8], and is commonly used for treating various malignant tumors in clinics [9,10,11,12,13]. Among its uses, it has a significant effect on lung cancer. According to traditional Chinese medicine, lung cancer is due to the accumulation of phlegm and clumping in the lungs. and the use of CPPP can dissolve phlegm and resolve nodules. A study investigated the clinical efficacy of CPPP adjuvant therapy in 74 patients with advanced non-small-cell lung cancer. The results showed that CPPP could effectively improve their prognosis and restore patients’ physical function [14].

CPPP contains various physiologically active compounds, including phenanthrene, bibenzyls, flavonoids, terpenoids, and glycosides [15]. According to previous studies, phenanthrene is the main active anti-tumor component of CPPP, including cremaphenanthrene L [16], blestriarene C [17], and shanciol H [18], which can inhibit the proliferation of tumor cells to varying degrees. At the same time, other important compounds with anti-tumor activity, except for phenanthrene, were ignored. Modern pharmacological studies have found that CPPP can inhibit the proliferation of tumor cells [19], induce the apoptosis of tumor cells [20], and interfere with the invasion and metastasis of tumor cells [21]. However, the mechanisms underlying this have not been discussed in detail. Furthermore, research has not differentiated between different commercial specifications of CPPP. Therefore, the pharmacodynamic material basis and action mechanisms of different commercial specifications of CPPP against tumors are still unclear, limiting its clinical applications and the development of modern pharmaceutical preparations.

It is well-known that TCM exerts efficacy through the synergistic action of multiple chemical components and targets. However, not all components in TCM exert specific effects, and many are ineffective, unknown, and even toxic, which affects TCM’s safety, quality controllability, and effectiveness. Effective ingredient combinations contain specific ingredients with precise contents, which can effectively solve the above problems. Analysis of the spectrum–effect relationship allows for understanding the correlations of the changes in chemical composition between different batches of medicinal materials with the actual pharmacological effects using various algorithmic models to screen the components related to specific pharmacological effects. This method not only excavates the pharmacological material basis of TCM, but also preserves its holistic and multi-component synergistic characteristics. In addition, most TCMs are administered orally, and the medicinal components enter the bloodstream through metabolism and absorption, thereby exerting an effect. Analyzing the components in the bloodstream can elucidate the actual medicinal effects of TCM. Through combining a spectrum–effect relationship analysis with serum pharmacochemistry, the effective ingredients of TCM entering the bloodstream can be screened, in order to form an effective ingredient combination with specific ingredients and precise contents, which can replace the original medicinal materials in application. This can also provide a scientific basis for the selection and breeding of excellent varieties and the quality evaluation of medicinal materials.

Autophagy and apoptosis are two regulated forms of cell death, and maintaining intracellular homeostasis depends on the interplay between apoptosis and autophagy [22]. Crucially, the AMP-activated protein kinase (AMPK)–mammalian target of the rapamycin (mTOR) signaling pathway plays an important bidirectional regulatory role in autophagy-dependent apoptosis [23]. However, it is unclear which target or protein of this pathway regulates apoptosis. BH3-only proteins exert a pro-apoptotic effect by activating Bax or Bak directly or indirectly [24]. In particular, we found in the preliminary RNA-seq results that ULK1 and BMF (a BH3-only protein), but not Bim, Bad, Bid, or other BH3-only proteins, responded to the changes in autophagy and apoptosis after drug administration. Therefore, we hypothesize that AMPK–mTOR regulates the interaction between autophagy and apoptosis through ULK1 and BMF.

In this study, UHPLC-MS fingerprints of MCG and BQZ were established separately and common components were identified. Inhibitory effects on the proliferative viability and migratory ability of A459 and H1299 non-small-cell lung cancer cells were evaluated as pharmacodynamic indicators. Grey relation analysis (GRA) and bivariate correlation analysis (BCA) were used to determine the spectrum–effect correlation. Next, the identification and analysis of components of herbal extracts, blank serum, and migration components of drug-containing serum were performed using UHPLC-Q-Exactive Orbitrap MS. The results of the two analyses were combined to jointly screen out the anti-lung-cancer candidate active monomers of CPPP. Then, all active monomers were prepared into a formulation with a clear chemical composition and anti-lung-cancer activity equivalent to that of the original medicinal materials. We further investigated its effects on the apoptosis and autophagy of tumor cells and whether these processes were related to the AMPK–mTOR–ULK1/BMF pathway. In summary, this study developed effective ingredient combinations with specific components and clear contents from CPPP that are expected to treat lung cancer and attempted to reveal the interaction between autophagy and apoptosis in tumor cells via the AMPK–mTOR–ULK1/BMF pathway.

## 2. Results

### 2.1. UHPLC-MS Fingerprint Similarity Analysis and Identification of Common Compounds

The precision, stability, and repeatability of the established fingerprints were assessed using the RSD (%) of the relative retention time (RRT) and relative peak area (RPA) to determine common chromatographic peaks. Specific results are presented in Table S2. Method validation indicated that the RRT-RSD (%) for instrument precision, method repeatability, and method stability over 24 h was below 1.00%, while the RPA-RSD (%) remained under 3.00%. This confirms that the UPLC fingerprinting method is reliable and stable within a 24 h period.

**Figure 1:**
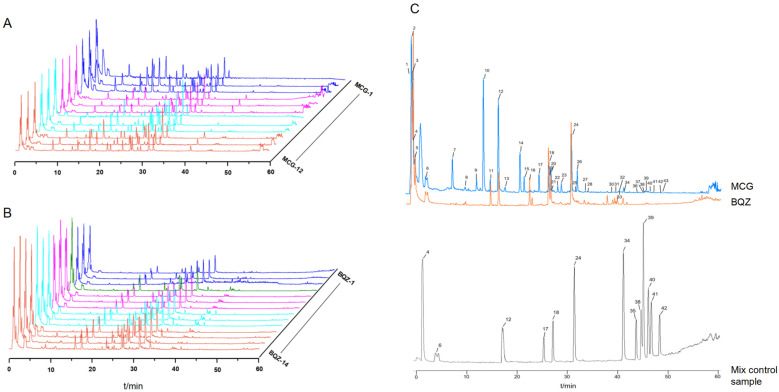
UHPLC-MS fingerprint and reference fingerprint. (**A**) Twelve batches of MCG samples. (**B**) Fourteen batches of BQZ samples. (**C**) Reference fingerprints of MCG, BQZ, and mixed control samples. Note: 4—malic acid, 6—gastrodin, 12—2-isobutylmalic acid, 17—loroglossin, 18—dactylorhin A, 24—militarine, 34—batatasin III, 35—2,7-dihydroxy-1-(4-hydroxybenzyl)-4-methoxyphenanthrene, 38—blestriarene A, 39—2-(*p*-hydroxybenzyl)-3′,5-dihydroxy-3-methoxybibenzyl, 40—blestriarene B, 41—monbarbatain A, and 42—coelonin.

MCG-1 and BQZ-1 were selected as control fingerprints, and fingerprints of various product specifications of CPPPs were established separately. An overlay of the fingerprints of 12 batches of MCG samples and 14 batches of BQZ samples was obtained (Figure 1A,B). A total of 43 common peaks were matched and identified, respectively, from MCG and BQZ (with each peak area accounting for more than 0.1% of the total peak area), and 12 components were identified according to the retention time of standard substances (Figure 1C). A total of 31 components (Table 1) were inferred using mass spectrometry. The identification results showed that the common components of MCG and BQZ were the same, but the contents in both of them were very different.

When using the control fingerprints of various product specifications of CPPPs, the similarity between 12 batches of MCG ranged from 0.925 to 0.945, and the similarity between 14 batches of BQZ ranged from 0.868 to 0.929 (Table S3). Among them, MCG only has one origin, *C. appendiculata*, indicating a good consistency in the chemical composition of this origin. Meanwhile, BQZ includes two types of origin, namely, *P. bulbocodioides* and *P. yunnanensis*, and the chemical consistency of these two origins is still good, probably because the two origins belong to the same genus of plants, indicating that there is little difference in chemical composition within the genus of *Pleione D. Don*. Nevertheless, the similarity between the fingerprints of 12 batches of MCG and BQZ control fingerprints was 0.670, 0.648, 0.665, 0.657, 0.629, 0.661, 0.674, 0.672, 0.633, 0.684, 0.671, and 0.692, respectively, indicating significant differences in chemical composition between MCG and BQZ.

### 2.2. Determination of Anti-Lung-Cancer Efficacy Indexes In Vitro

The proliferation process of tumor cells is a major foundation for the onset and progression of cancer, and the spread and metastasis of cancer are determined by the migratory and invasive capabilities of these malignant cells. The CCK-8 method is usually used to measure the cell proliferation ability, while the scratch assay is used to measure the cell migration ability, in order to evaluate the anti-tumor activity of drugs.

**Figure 2:**
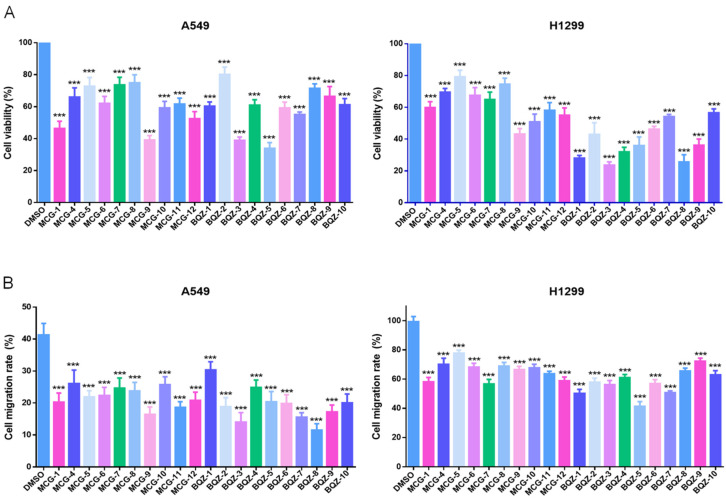
The effect of 10 batches of MCG samples and 10 batches of BQZ samples on the anti-lung-cancer activities in vitro. (**A**) The effect on the proliferation activity of A549 and H1299 cells. (**B**) The effect on the migration ability of A549 and H1299 cells. Data are mean ± SD, *n* = 6, *** *p* < 0.001, the treatment group vs. the control.

The MCG-1 of the CPPP sample was diluted with a medium into eight concentrations ranging from 0.125 mg/mL to 3 mg/mL, and its effect on the proliferative vitality of A549 and H1299 cells was assessed. The results show that the inhibitory effect of MCG-1 on lung cancer cells was dose-dependent and time-dependent, and the IC_50_ of the A549 and H1299 cells at 48 h was 1.021 mg/mL and 0.679 mg/mL, respectively (Figure S2). Thus, we chose 1 mg/mL as the final concentration to determine the inhibitory rate of the 20 CPPP samples. As shown in Figure 2A, the inhibitory effects of 20 CPPPs on lung cancer cells were quite different. Subsequently, we chose a drug concentration without cytotoxicity to measure cell migration activity. As shown in Figure 2B, the 20 CPPPs could inhibit the migration of lung cancer cells to varying degrees. The results of the in vitro pharmacodynamic indexes of the 20 batches of CPPPs are shown in Table S4. The anti-tumor activity of BQZ was slightly better than that of MCG.

### 2.3. Spectrum–Effect Correlation Analysis

#### 2.3.1. GRA Analysis

In this study, the common peak areas of 10 MCG and 10 BQZ fingerprints were used as one set of variables, and the inhibition rates of the A549 and H1299 cells on proliferation and migration were used as another set of variables. The results of GRA are shown in Supplementary Tables S5 and S6. The value of the correlation coefficient of ≥0.8 indicates that the two variables were significantly correlated. For MCG, the grey correlation coefficients between the area of their common peaks and the cell proliferation inhibition rates of the A549 and H1299 cells were 0.592–0.871 and 0.576–0.869, respectively. The grey correlation coefficients between the area of common peaks and the cell migration inhibition rates of the A549 and H1299 cells were 0.603–0.884 and 0.583–0.893, respectively. Overall, the peaks with high grey correlation coefficients included P12 (2-isobutylmalic acid), P34 (batatasin III), P35 (2,7-dihydroxy-1-(4-hydroxybenzyl)-4-methoxyphenanthrene), P6 (gastrodin), P42 (coelonin), P41 (monbarbatain A), and P25 (gymnoside III), etc. For BQZ, the grey correlation coefficients between the area of common peaks and the cell proliferation inhibition rates of the A549 and H1299 cells were 0.638–0.877 and 0.618–0.887, respectively. The grey correlation coefficients between the area of common peaks and the cell migration inhibition rates of the A549 and H1299 cells were 0.603–0.929 and 0.629–0.93, respectively. Overall, the peaks with high grey correlation coefficients included P34 (batatasin III), P24 (militarine), P38 (blestriarene A), P35 (2,7-dihydroxy-1-(4-hydroxybenzyl)-4-methoxyphenanthrene), P39 (2-(*p*-hydroxybenzyl)-3′,5-dihydroxy-3-methoxybibenzyl), and P6 (gastrodin). As a result, the above substances were deduced to possess anti-lung-cancer activity.

#### 2.3.2. BCA Analysis

In BCA analysis, the larger the absolute value of the correlation coefficient of the component peak, the greater the influence of the ingredient on the anti-lung-cancer activity. The BCA analysis results are shown in Supplementary Tables S7 and S8. According to the correlation coefficients, for MCG, the anti-lung-cancer ability significantly correlated with P34 (batatasin III), P12 (2-isobutylmalic acid), P6 (gastrodin), P42 (coelonin), P15 (coelovirin B), P35 (2,7-dihydroxy-1-(4-hydroxybenzyl)-4-methoxyphenanthrene), P25 (gymnoside III), and P41 (monbarbatain A), etc., in a positive way. For BQZ, the anti-lung-cancer ability significantly correlated with P24 (militarine), P38 (blestriarene A), P35 (2,7-dihydroxy-1-(4-hydroxybenzyl)-4-methoxyphenanthrene), P42 (coelonin), P34 (batatasin III), P20 (gymnoside), P5 (citric acid), etc., in a positive way.

The singular application of statistical methods proves inadequate for precisely identifying the effective components in CPPP. Consequently, this experiment integrated two mathematical statistical approaches to collaboratively screen for active constituents, thereby enhancing the accuracy of the results. Under various anti-lung-cancer indicators, we can use the intersection of the components’ peaks with a correlation coefficient and a positive bivariate correlation coefficient ranking in the top ten to obtain the effective anti-lung-cancer components of this indicator. Various indicators were comprehensively screened to obtain candidates for effective ingredient groups with the anti-lung-cancer effects of MCG and BQZ (Table 2). All results indicate that 13 ingredients may have a high relativity regarding the anti-lung-cancer activity of MCG, which mainly includes phenanthrene components such as blestriarene B, coelonin, monbarbatain A, and 2,7-dihydroxy-1-(4-hydroxybenzyl)-4-methoxyphenanthrene; bibenzyl components such as batatasin III; glycoside components such as gymnoside III, coelovirin B, grammatophylloside B, etc.; and the basic structural units of glycoside components, 2-isobutylmalic acid and gastrodin. These may form the main pharmacological substance basis for the anti-lung-cancer activity of MCG. Similarly, 12 ingredients in BQZ also have a high correlation with anti-lung-cancer activity, which mainly include phenanthrene components such as 2,7-dihydroxy-1-(4-hydroxybenzyl)-4-methoxyphenanthrene, coelonin, and blestriarene A; bibenzyl components such as batatasin III and 2-(*p*-hydroxybenzyl)-3′,5-dihydroxy-3-methoxybibenzyl; organic acid components such as malic acid and citric acid; glycoside components such as militarine, dactylorhin A, gymnoside VI, etc.; and the basic structural units of glycoside components, 2-isobutylmalic acid and gastrodin. These may form the main pharmacological substance basis for the anti-lung-cancer activity of BQZ.

### 2.4. UHPLC-Q-Exactive Orbitrap MS Analysis of Mice Serum

**Figure 3:**
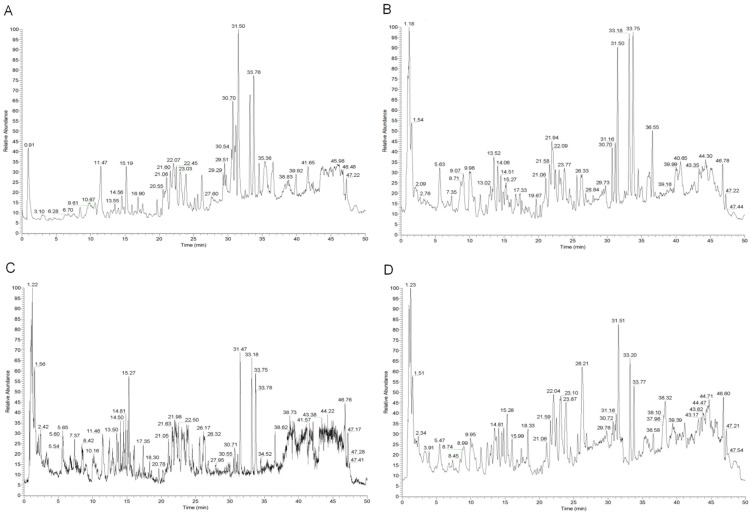
Total ion chromatograms (TICs) of serum samples from mice in the negative ion mode with lung cancer. (**A**) Blank serum, (**B**) model serum, (**C**) serum of MCG, and (**D**) serum of BQZ.

In this study, UPLC-Q-Exactive Orbitrap MS was employed to characterize and identify components in serum samples from mice with lung cancer after oral administration. TIC chromatograms of the blank serum, model serum, and drug serum under the negative ion mode are shown in Figure 3. Through a comparison and analysis of the primary and secondary mass spectrometry data of the model serum and drug serum, combined with mass spectrometry data from our previous studies and references, 33 components were identified in the drug serum, including 21 absorption prototype components (Table 3) and 17 metabolites (Table 4).

The prototype components mainly include 14 organic acid components such as malic acid, citric acid, and 2-isobutyl malic acid, etc.; glucosyloxybenzyl succinate derivatives such as dactylorhin C, gymnoside, and militarine, etc.; as well as gastrodin. Glucosyloxybenzyl succinate derivatives were highly abundant in the alcoholic extracts of CPPPs, but their abundance was very low in the mice serum. This kind of component is composed of gastrodin and 2-isobutylmalic acid or other organic acids as unit structures linked by ester bonds, showing a poor stability. Therefore, it was speculated that the ester bond was hydrolyzed and converted into gastrodin and organic acids such as 2-isobutylmalic acid when absorbed into the blood. The metabolic pathway is shown in Figure S3. Therefore, gastrodin, 2-isobutylmalic acid, 2-isobutyltartaric acid, 2-benzylmalic acid, and eucomic acid were partially introduced into the blood as prototypes, and some of them were probably hydrolyzed products of glycosides in the medicinal materials. Additionally, a total of 10 phenanthrene and bibenzyl components were found, all of which were present in the serum while binding single or multiple glucuronic acids ([M + C_6_H_7_O_6_]^−^, [M + C_12_H_15_O_12_]^−^).

Except for a few components, such as coelovirin B, bulbocodioidins G, and 2,7-dihydroxy-4-methoxyphene, most were only found in the serum samples of MCG. Meanwhile, *o*-hydroxybenzoic acid, *M − H*ydroxybenzoic acid, and dactylorhin E were only found in the serum samples of BQZ, while the other 27 components were present in the serum samples.

Combined with the spectrum–effect relationship analysis and serum pharmacochemistry, 11 candidate active ingredients were selected from two different commercial specifications of CPPPs, MCG and BQZ, including monbarbatain A, 2,7-dihydroxy-1-(4-hydroxybenzyl)-4-methoxyphenanthrene, coelonin, blestriarene A, blestriarene B, 2-(*p*-hydroxybenzyl)-3′,5-dihydroxy-3-methoxybibenzyl, batatasin III, gastrodin, 2-isobutylmalic acid, malic acid, and citric acid. The above ingredients has significant differences in content between MCG and BQZ. Hence, different commercial specifications of CPPPs may exert anti-tumor effects through different proportions of candidate active ingredients to form effective ingredient combinations.

### 2.5. Validation of the Anti-Lung-Cancer Activities of Candidate Active Monomers In Vitro

To validate the reliability of the results, the effects of 11 candidate monomer components on the proliferation activity and migration ability for lung cancer cells were further evaluated, as shown in Figure S4. The results of the proliferation and migration abilities of the positive drug cisplatin for treating lung cancer on A549 and H1299 cells are shown in Figures S5 and S6. The results showed that the 11 monomers of the candidate effective ingredient group had varying inhibitory effects on the proliferation activity and migration ability of lung cancer cells. Among them, the phenanthrene ingredients showed excellent activity in inhibiting the proliferation and migration ability of lung cancer cells, which are polycyclic aromatic hydrocarbons containing three fused benzene rings. The efficacy of monbarbatain A and blestriarene B was equivalent to or even greater than the positive control. The effectiveness of bibenzyl ingredients was slightly worse than that of phenanthrenes and exhibited a significant inhibition of the proliferation activity and migration ability of lung cancer cells at 50 µM. Furthermore, gastrodin and organic acids could only inhibit the proliferation and migration of lung cancer cells to a certain extent at high concentrations, which is consistent with existing research findings [25].

### 2.6. Validation of the Anti-Lung-Cancer Activities of Candidate Effective Ingredient Combinations In Vitro and In Vivo

**Figure 4:**
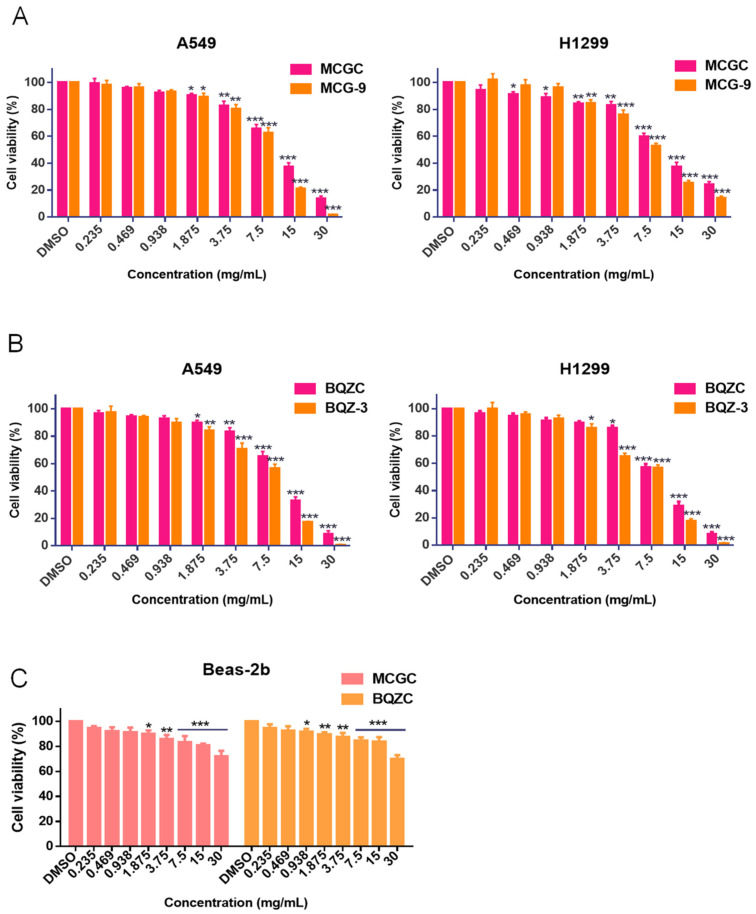
The anti-lung-cancer activities of MCGC, MCG-9, BQZC, and BQZ-3 in vitro. (**A**) The effects of MCGC and MCG-9 on the proliferation of A549 and H1299 cells. (**B**) The effects of BQZC and BQZ-3 on the proliferation of A549 and H1299 cells. (**C**) The effects of MCGC and BQZC on the proliferation of Beas-2b cells. Data are mean ± SD, *n* = 6, * *p* < 0.05, ** *p* < 0.01, and *** *p* < 0.001, the treatment group vs. the control.

MCG-9 and BQZ-3 showed a prominent and comprehensive anti-lung-cancer efficacy in vitro. Therefore, according to the content and proportion of 11 active monomer components in crude MCG-9 and BQZ-3 (30 mg/mL), the anti-lung-cancer candidate effective ingredients of MCG and BQZ were combined, respectively, which are referred to as MCGC and BQZC. The content and proportion of each monomer of MCG and BQZ are shown in Tables S9 and S10 (since most of the glycosidic components in the CPPPs were hydrolyzed by digestive tract enzymes and then entered into the blood with 2-isobutylmalic acid and gastrodin to exert drug effects, the contents of gastrodin and 2-isobutylmalic acid in the candidate effective ingredient combinations were their contents after alkaline hydrolysis of the original medicinal materials) [26]. As displayed in Figure 4A,B, with the same dosage of crude medicinal herbs, MCGC, MCG-9, BQZC, and BQZ-3 all inhibited the proliferation activity of lung cancer cells in a dose-dependent manner, and the anti-tumor activities of effective ingredient combinations of different commercial specifications of CPPPE were similar to CPPPE. The IC_50_ values of MCGC for A549 and H1299 cells were 10.56 mg/mL and 10.5 mg/mL; the IC_50_ values of MCG-9 for A549 and H1299 cells were 8.402 mg/mL and 7.778 mg/mL; the IC_50_ values of BQZC for A549 and H1299 cells were 9.814 mg/mL and 9.762 mg/mL; and the IC_50_ values of BQZ-3 for A549 and H1299 cells were 6.821 mg/mL and 6.594 mg/mL, respectively. The results indicated that MCGC and BQZC can represent the anti-lung-cancer activity of the original medicinal materials in vitro. Simultaneously, we conducted an assessment of the cytotoxicity of MCGC and BQZC on the human normal cell line Beas-2b (Figure 4C), and the IC50 values exceeded 30.0 mg/mL, indicating that their toxicity toward normal cells is lower than that in cancer cells.

**Figure 5:**
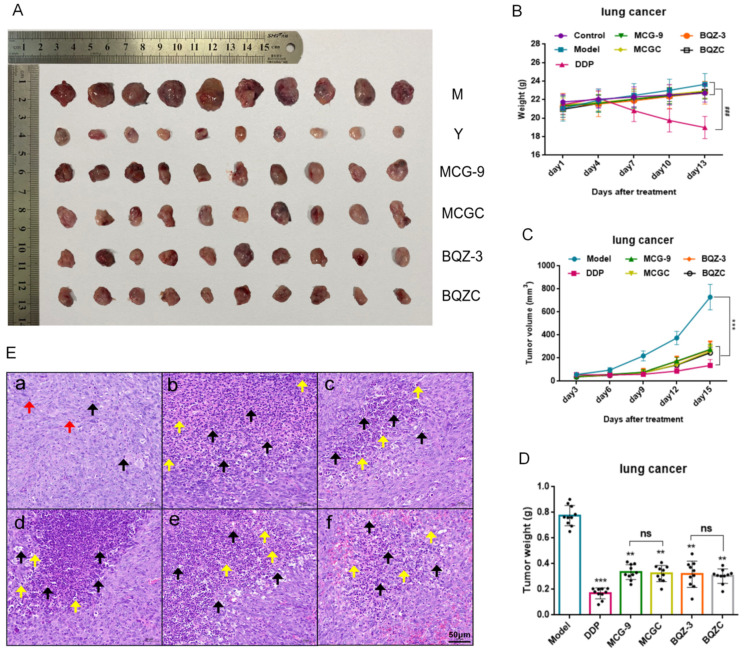
The effect of MCG-9, MCGC, BQZ-3, BQZC, and positive drugs on the growth of an LLC xenografted tumor in vivo. (**A**) Tumor images. (**B**) The body weight was measured every three days. (**C**) Tumor growth curves. (**D**) Tumor weight. (**E**) Tumor histopathological morphology (HE staining, ×200, *n* = 3). (**a**)—model group; (**b**)—positive drug group; (**c**)—MCG-9 group; (**d**)—MCGC group; (**e**)—BQZ-3 group; and (**f**)—MCGC group. Note: the red arrow indicates the mitotic phase, the black arrow indicates necrotic cells, and the yellow arrow indicates infiltration of inflammatory cells. Data are presented as mean ± SD, ** *p* < 0.01, and *** *p* < 0.001 vs. model group; ### *p* < 0.001 vs. the control group; ns, not significant.

To evaluate the anti-lung-cancer efficacy of MCGC and BQZC in vivo, stable subcutaneous xenograft tumor models for lung cancer were established using LLC cells. The excised tumors from each group are depicted in Figure 5A. MCGC, BQZC, and the extracts of their raw medicinal materials significantly inhibited tumor growth by reducing the average tumor volume and weight (*p* < 0.01, *p* < 0.05, and *p* < 0.001), and there were no significant differences between the MCGC and BQZC groups and their respective extracts of raw medicinal material groups (*p >* 0.05) (Figure 5C,D). The tumor inhibition rates of each group are shown in Table S11. In the lung cancer model, the efficacy of the cisplatin group was slightly better than that of the other treatment groups, but the weight of the mice in the cisplatin group was significantly reduced compared to those in the normal group and model group (Figure 5B), and their survival status was poor. As depicted in Figure 5E, compared with the model group, the positive drug group and the MCGC, MCG-9, BQZC, and BQZ-3 groups showed extensive tumor cell necrosis, nuclear fragmentation, and the deep staining or disappearance of solid shrinkage in the field of vision (as indicated by the black arrow). Many inflammatory cells can be seen infiltrating the necrotic area (as indicated by the yellow arrow). Nuclear dysplasia was low, and mitotic figures were rare. These results suggested that all groups could inhibit tumor cell division and induce tumor tissue inflammation and necrosis in tumor-bearing mice.

### 2.7. BQZC Regulates Autophagy and Apoptosis Cross-Talk to Inhibit Lung Cancer Cell Growth

**Figure 6:**
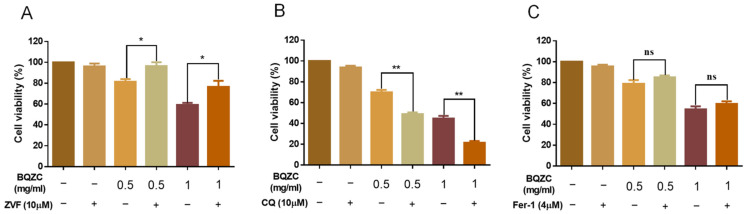
The effect of BQZC alone or combined with different cell death inhibitors on the cell viability of tumor cells. A549 cells were treated with BQZC with or without (**A**) ZVF, (**B**) CQ, and (**C**) Fer-1 for 48 h, and cell viability was detected. Data are presented as mean ± SD, * *p* < 0.05, ** *p* < 0.01. ns, not significant.

We selected BQZC, which has a better anti-tumor efficacy, to further explore the possible mechanism of its anti-lung-cancer effect. In order to evaluate the main cell death program induced by BQZC, we used specific inhibitors, including Z-VAD-FMK (ZVF, pan-caspase inhibitor), chloroquine (CQ, autophagy inhibitor), and Ferrostatin (Fer-1, ferroptosis inhibitor). The results showed that Z-VAD-FMK could significantly rescue BQZC-induced cell death in A549 cells (Figure 6A). A statistically significant decrease in the viability of the A549 cells was observed when CQ and BQZC were administered together compared to when only BQZC was used (Figure 6B). However, Fer-1 could not remarkably inhibit the cell death caused by BQZC (Figure 6C). These results proved that BQZC might inhibit tumor cell growth by regulating the interaction between autophagy and apoptosis.

#### 2.7.1. BQZC Induces Lung Cancer Cell Apoptosis In Vitro and In Vivo

**Figure 7:**
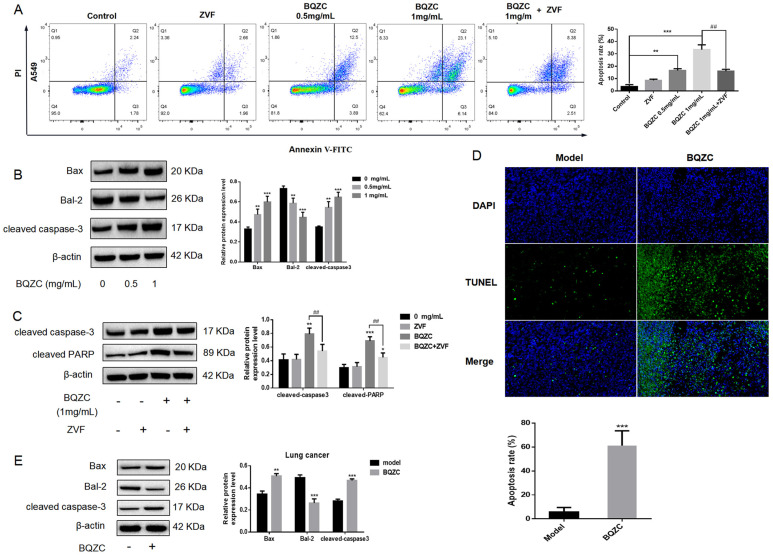
BQZC induces apoptosis in A549 cells and lung tumor tissues. (**A**) The apoptotic ratio of A549 cells was measured using flow cytometer. (**B**) The expression levels of apoptosis-related proteins, including Bax, Bcl-2, and cleaved caspase-3, were assessed in cells treated with BQZC using Western blot analysis. β-Actin served as a loading control. (**C**) A549 cells were treated with or without ZVF for 24 h in the presence of BQZC, followed by Western blot detection of cleaved caspase-3 and cleaved PARP expression levels. (**D**) Apoptosis within lung tumor tissue was quantified utilizing the TUNEL assay, highlighting apoptotic cells (green) alongside cell nuclei (blue). (**E**) The expression of apoptosis-related proteins in tumor tissues was further analyzed through Western blot. β-actin was employed as a loading control. All data are presented as mean ± SD, *n* = 3 independent experiments, * *p* < 0.05, ** *p* < 0.01, and *** *p* < 0.001 vs. the control, ## *p* < 0.01 vs. BQZC treatment.

To elucidate the impact of BQZC on apoptosis, a flow cytometric analysis was conducted on A549 cells utilizing annexin V-FITC/PI. As illustrated in Figure 7A, treatment with BQZC markedly increased the proportion of apoptotic cells in a dose-dependent manner. In addition, Western blot analysis demonstrated that BQZC administration significantly increased the protein levels of Bax and cleaved caspase-3 in a dose-dependent manner. In contrast, a reduction in the expression of the anti-apoptotic protein Bcl-2 was observed (Figure 7B). ZVF could suppress cleaved caspase-3. The combination of ZVF and BQZC pretreatment significantly rescued BQZC-induced apoptosis (Figure 7A) and markedly reduced the expression levels of cleaved caspase-3 and cleaved caspase-PARP (Figure 7C). The xenograft model was employed to further elucidate the impact of BQZC-induced apoptosis on tumor growth in vivo. TUNEL assays were conducted to investigate the effects on tumor cell apoptosis. As illustrated in Figure 7D, an enhancement in green fluorescence was observed in the BQZC treatment group. Additionally, Western blot analysis was performed to assess the expression levels of Bax, Bcl-2, and cleaved caspase-3 within the tumors. We observed increased expressions of Bax and cleaved caspase-3, while there was a significant reduction in Bcl-2 protein levels (Figure 7E).

#### 2.7.2. BQZC Induces Lung Cancer Cell Autophagy In Vitro and In Vivo

**Figure 8:**
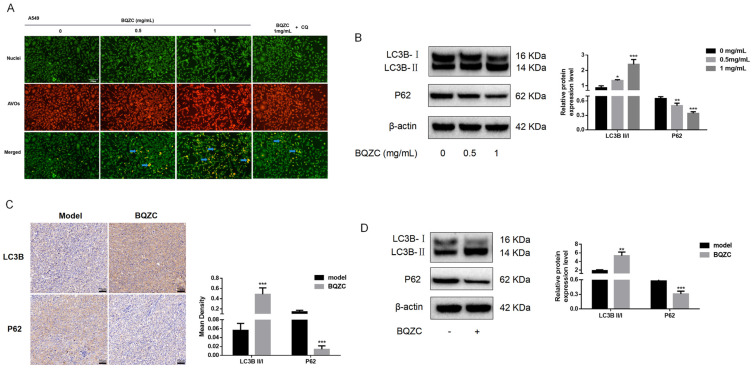
BQZC induces autophagy in A549 cells and lung tumor tissues. (**A**) Fluorescence microscopic images of A549 cells treated with BQZC and stained with acridine orange for the detection of AVOs. The cytoplasmic and nuclear regions exhibited green fluorescence, while the AVOs radiated a red hue. Arrows denote the presence of AVOs (scale bar: 100 μm; magnification, ×200). (**B**) Western blot analysis assessed the expression levels of autophagy-related proteins, including LC3B-II/I and p62, in cells treated with BQZC. β-Actin served as a loading control. (**C**) IHC analysis of tumor tissues was used to assess the expression levels of LC3I/II and p62 across various experimental groups. (**D**) The expression levels of autophagy-related proteins within tumor tissues were examined through Western blotting. β-actin served as a loading control. All data are presented as mean ± SD, *n* = 3 independent experiments, * *p* < 0.05, ** *p* < 0.01, and *** *p* < 0.001 vs. the control.

As previously demonstrated, inhibiting autophagy enhances the pro-apoptotic effect of BQZC. Therefore, clarifying the effect of BQZC on autophagy within lung cancer cells is essential for elucidating the relationship between autophagy and apoptosis. We conducted AO staining experiments to ascertain whether BQZC treatment induced autophagy through the formation of AVOs. Notably, compared to the control group, there was a marked increase in AVO formation in the BQZC-treated group (Figure 8A). Furthermore, we assessed the expression levels of the autophagy markers LC3 and p62 using Western blot analysis. The results indicated that BQZC significantly increased the LC3B-II/I ratio in a dose-dependent manner while simultaneously reducing p62 levels (Figure 8B). In vivo, we employed immunohistochemistry and Western blotting to evaluate the expression of key proteins, including the LC3B-II/I ratio and p62. These findings were consistent with in vitro data, demonstrating that BQZC treatment led to an increase in the LC3B-II/I ratio and a decrease in p62 levels (Figure 8C,D).

#### 2.7.3. Inhibition of Autophagy Promotes BQZC-Induced Apoptosis in Lung Cancer Cells

**Figure 9:**
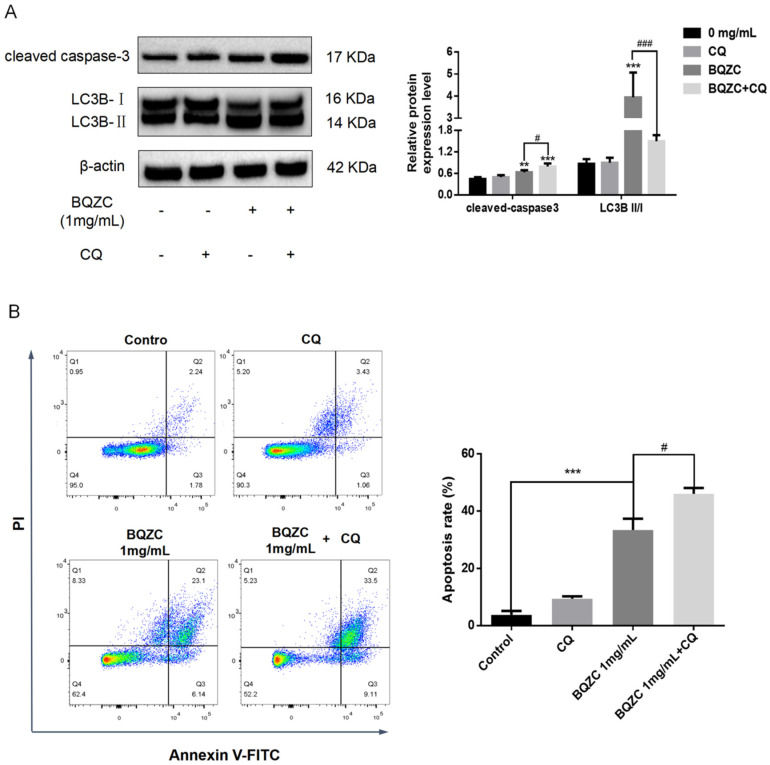
The suppression of autophagy enhances the apoptosis of A549 cells induced by BQZC. (**A**) A549 cells were treated with either BQZC alone or in combination with CQ for 24 h, followed by Western blot analysis to assess the expression levels of cleaved caspase-3 and LC3B-II/I. β-actin served as a loading control. (**B**) The analysis of cell apoptosis was conducted using flow cytometry. All data are presented as mean ± SD, *n* = 3 independent experiments, ** *p* < 0.01, and *** *p* < 0.001 vs. the control, # *p* < 0.05 and ### *p* < 0.001 vs. BQZC treatment.

There is compelling evidence indicating that autophagy can either magnify or eliminate the apoptotic effects of anti-tumor drugs in cancer cells [27]. In order to clarify the effect of autophagy in BQZC-induced cellular apoptosis, CQ was employed to inhibit this process. The results from Western blot analysis revealed that, compared to cells treated solely with BQZC, CQ upregulated the expression of cleaved caspase-3 in cells subjected to co-treatment with BQZC while concurrently downregulating the LC3B-II/I ratio (Figure 9A). Coherently, flow cytometry analysis demonstrated that adding CQ markedly increased the percentage of apoptotic lung cancer cells following BQZC treatment (Figure 9B). Consequently, the combination treatment of BQZC and CQ augmented the apoptotic response in lung cancer cells, indicating that, while BQZC induced the apoptosis of lung cancer cells, autophagy induced by BQZC also inhibited apoptosis to a certain extent.

#### 2.7.4. AMPK–mTOR–ULK1/BMF Pathway Was a Bridge Between Autophagy and Apoptosis in Lung Cancer Cells In Vitro and In Vivo

**Figure 10:**
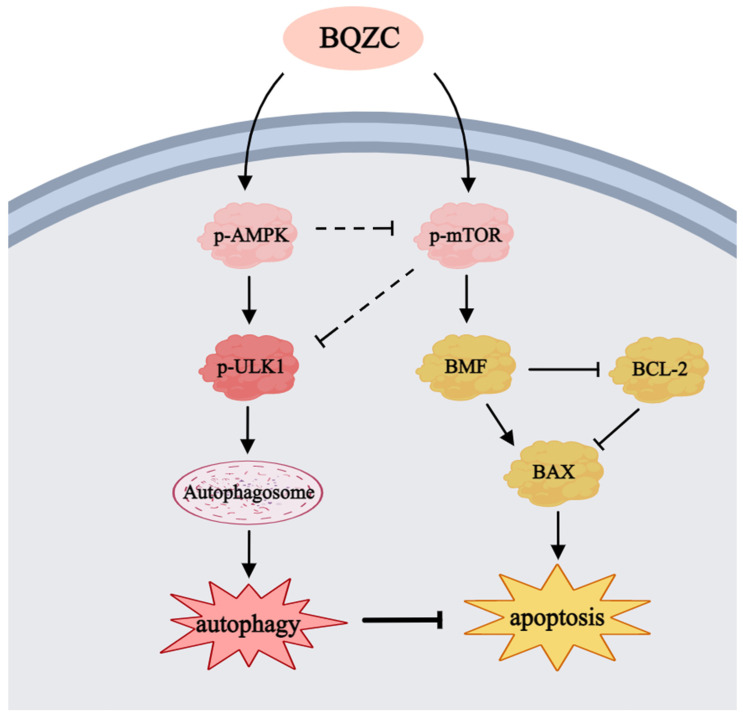
A schematic of BQZC-induced apoptosis and autophagy in lung cancer cells. BQZC induced apoptosis in A549 cells by activating the intrinsic pathway mediated through mTOR–BMF–Bax signaling cascades, simultaneously inducing autophagy via AMPK–ULK1 signaling. Arrows denote activation, whereas bars signify inhibition.

**Figure 11:**
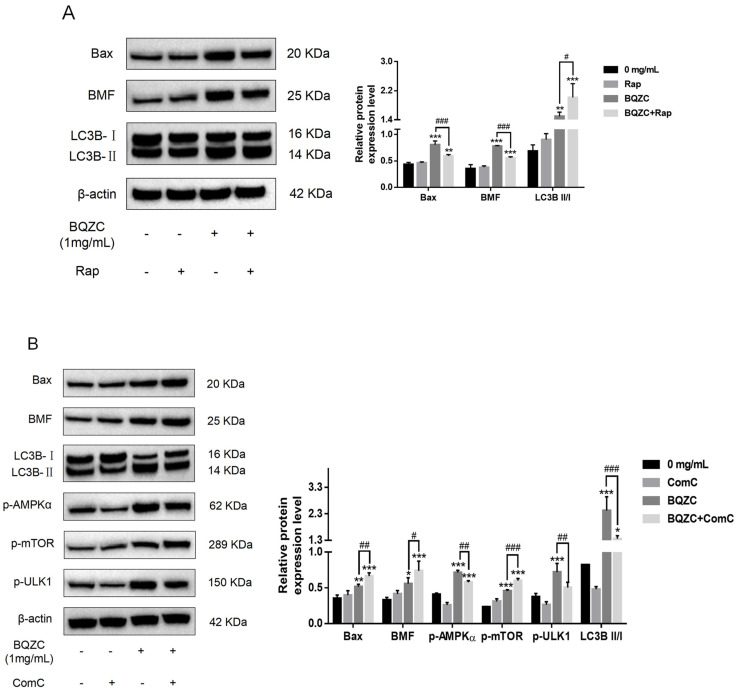

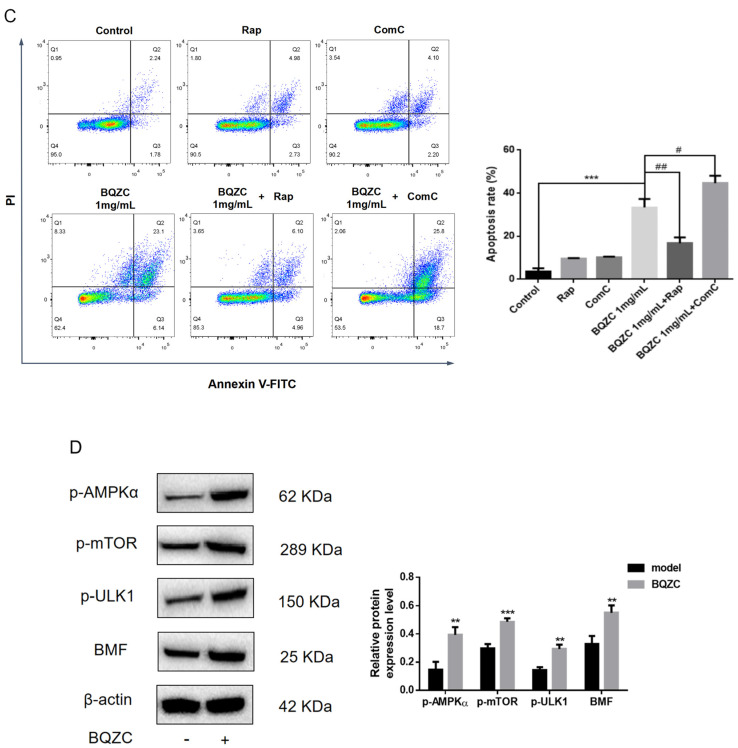
The BQZC-activated AMPK–mTOR–ULK1/BMF pathway is pivotal in BQZC-induced autophagy-dependent apoptosis in lung cancer cells. (**A**) The A549 cells were treated with BQZC, both in the presence and absence of Rap, for 24 h. Subsequently, the expression levels of Bax, BMF, and LC3B-II/I were assessed utilizing Western blot analysis. (**B**) The A549 cells were treated with BQZC, both in the presence and absence of ComC, for 24 h. Subsequently, the expression levels of Bax, BMF, and LC3B-II/I and the protein expression levels of AMPK–mTOR–ULK1 phosphorylation were assessed utilizing Western blot analysis. β-actin served as a loading control. (**C**) The impact of Rap and ComC on BQZC-induced apoptosis is shown, and cell apoptosis rates were measured using flow cytometry. (**D**) The expression of BMF, p-AMPK, p-mTOR, and p-ULK1 in tumor tissues was detected using Western blotting. β-actin was used as a loading control. All data are presented as mean ± SD, *n* = 3 independent experiments, * *p* < 0.05, ** *p* < 0.01, *** *p* < 0.001 vs. the control, # *p* < 0.05, ## *p* < 0.01, ### *p* < 0.001 vs. BQZC treatment.

The interplay between autophagy and apoptosis induced by BQZC has been substantiated previously, but it is still unclear through which target or pathway this effect is transmitted. With reference to the literature [23,24] and transcriptomics, we predicted that AMPK–mTOR–ULK1/BMF might serve as a crucial mediator in the relationship between BQZC-induced autophagy and apoptosis. Therefore, in this study, we confirmed that BQZC, one the one hand, activated the mTOR–BMF–Bax signaling pathway to promote lung cancer cell apoptosis, and on the other, induced autophagy by the AMPK–ULK1 signaling pathway to resist excessive apoptosis. The negative regulation of AMPK on mTOR may be the intrinsic reason why autophagy inhibits apoptosis (Figure 10). To confirm the above viewpoint, we pretreated A549 cells with Rap (an inhibitor of mTOR) and ComC (an AMPK inhibitor), subsequently assessing the expressions of relevant proteins. Compared with the treatment of BQZC alone, Rap co-treated with BQZC activated the autophagy process, significantly enhanced the level of the LC3B-II/I ratio, and reduced the downstream protein BMF, thereby inhibiting apoptosis-related protein Bax (Figure 11A). On the other hand, ComC significantly attenuated the increase in p-AMPKα, p-ULK1, and the ratio of LC3B-II/I induced by BQZC, while concurrently enhancing the expression levels of p-mTOR, BMF, and Bax (Figure 11B). Furthermore, a flow cytometry analysis was conducted (Figure 11C), revealing that Rap and ComC distinctly reduced or elevated BQZC-induced apoptosis in a significant manner, respectively. Simultaneously, we performed a parallel analysis of the levels of p-AMPK, p-mTOR, p-ULK1, and BMF in tumor tissues via Western blotting. As depicted in Figure 11D, the expression levels of the above protein were significantly elevated in mice treated with BQZC, which indicated that BQZC simultaneously promoted the autophagy and apoptosis of mice lung cancer cells through the AMPK–mTOR–ULK1/BMF signaling pathway. These results imply that the interplay between autophagy and apoptosis induced by BQZC is mediated by the AMPK–mTOR–BMF axis.

## 3. Discussion

Considering that China ranks first in the world for both new lung cancer cases and deaths, the prevention and treatment of lung cancer still remain major clinical challenges. As such, it is important to develop TCM for anti-tumor effects with a low toxicity and high efficacy. Nevertheless, TCM comprises a multitude of ineffective and unidentified ingredients. This complexity poses significant challenges in elucidating their active substances and mechanisms, as well as in ensuring the stability of their quality [28]. Thus, it is imperative to develop effective ingredient combinations of TCM with definite components representing the efficacy of the original medicinal materials. In this study, we proposed an innovative method for identifying effective ingredient combinations of CPPP based on the spectrum–effect relationship combined with serum pharmacochemistry.

Various algorithm models are usually applied in the spectrum–effect relationship analysis of TCM [29]. GRA can score the similarity between the peak of the component and the trend of the pharmacodynamic index, in order to obtain the component with a high correlation with the pharmacodynamic index. This method can analyze the correlation degree of the factors and has few requirements for sample quality. BCA can explore the correlation between the data from an independent perspective by predicting and explaining the linear relationship between two variables. This method can be used for data with both a normal and non-normal distribution, and the independence and integrity of the data are preserved. GRA and BCA were combined in this study. The results showed that the GRA and BCA methods have a high consistency and can mutually confirm and supplement each other, making results more accurate.

For two different commercial specifications of CPPP—namely, MCG and BQZ—the identification results of common peaks of the UPLC-MS fingerprint showed that the number and types of common peaks were the same, but the content and proportion of each chemical component were significantly different. The pharmacological activities of BQZ and MCG against lung cancer in vitro and in vivo were almost the same, with BQZ being slightly better than MCG, but there was no significant difference. Combined with the spectrum–effect relationship analysis and serum pharmacochemistry, 11 active ingredients were selected from MCG and BQZ, including monbarbatain A, 2,7-dihydroxy-1-(4-hydroxybenzyl)-4-methoxyphenanthrene, blestriarene A, blestriarene B, coelonin, 2-(*p*-hydroxybenzyl)-3′,5-dihydroxy-3-methoxybibenzyl, batatasin III, gastrodin, 2-isobutylmalic acid, malic acid, and citric acid. Hence, different commercial specifications of CPPPs may exert anti-lung-cancer effects by utilizing different proportions of active ingredients. The reasonableness of using different sources of the CPPP as the same medicine was preliminarily explained.

This study, for the first time, confirmed the anti-lung-cancer activity of two components, namely 2,7-dihydroxy-1-(4-hydroxybenzyl)-4-methoxyphenanthrene and 2-(*p*-hydroxybenzyl)-3′,5-dihydroxy-3-methoxybibenzyl. Among the effective ingredient combinations of CPPP, phenanthrenes showed strong anti-tumor activity, consistent with previous reports [30,31]. Monbarbatain A was the most remarkable. At a low dose, monbarbatain A and blestriarene B began to exhibit activity in inhibiting tumor cell proliferation and migration, and their anti-lung-cancer activity was similar to that of the positive drugs, indicating that monbarbatain A and blestriarene B may be important markers of the anti-lung-cancer effects of MCGC and BQZC. This may be related to the structure of their dimeric phenanthrene. Following closely behind were 2,7-dihydroxy-1-(4-hydroxybenzyl)-4-methoxyphenanthrene and blestriarene A, whose structures are mono-substituted phenanthrene and dimeric dihydrophenanthrene. Coelonin is mono-substituted dihydrophenanthrene, whose anti-lung-cancer activity was the worst. Batatasin III and 2-(*p*-hydroxybenzyl)-3′,5-dihydroxy-3-methoxybibenzyl are bibenzyls, which are another class of anti-lung-cancer active ingredients in CPPP [32,33]. There is scarce research on this kind of component. In addition, gastrodin and organic acids could only inhibit the proliferation and migration of tumor cells to a certain extent at high concentrations, which is consistent with existing research findings [25]. Nevertheless, CPPP contains a higher composition of organic acids, and many glycosides are mostly hydrolyzed into gastrodin and 2-isobutylmalic acid when entering the bloodstream to exert pharmacological effects. Therefore, organic acids and gastrodin also contribute to the anti-lung-cancer effect of CPPP.

Regarding the mechanisms of action, we selected BQZC, which had a better anti-lung-cancer efficacy, to further explore the possible mechanism of its anti-lung-cancer effect. Autophagy is a highly conserved metabolic process prevalent in eukaryotic cells which involves the transport of intracellular substances to lysosomes for degradation as the predominant intracellular degradation pathway [34]. Notably, autophagy plays an essential role in the pathological processes of cancer and has been extensively investigated as a promising therapeutic target for anticancer therapy [34]. Specifically, LC3 is generally considered to be a marker of autophagy, and cytoplasmic LC3 (LC3-I) enzymatically hydrolyzes a small segment of polypeptide before transforming into membrane-bound LC3 (LC3-II) during autophagosome formation, with the ratio of LC3-II/I being utilized to assess the level of autophagy [35]. Meanwhile, p62, a multi-domain protein, binds to LC3 on the membrane of autophagic vesicles and recruits autophagic degradation substrates into autophagic vesicles to complete the degradation process. Therefore, the protein levels of p62 are often used to indicate the status of autophagic degradation [36]. In this study, we observed that the generation of AVOs in lung cancer cells induced by BQZC exhibited a concentration-dependent escalation. Furthermore, the expression levels of LC3-II and p62, both pivotal indicators of autophagic progression, demonstrated a tendency to increase and decrease, respectively.

As a proactive mechanism of programmed cell death, apoptosis is indispensable in maintaining homeostasis within the body [37]. The Bcl-2 protein family, encompassing pro-apoptotic members like Bax and anti-apoptotic members like Bcl-2, primarily regulates the intrinsic apoptotic pathways [38]. At the same time, the Bax/Bcl-2 ratio is a pivotal determinant in the induction of cellular apoptosis [39]. Caspases, a distinguished family of proteolytic enzymes, play an indispensable role in orchestrating the apoptotic process. Among them, caspase-3 functions as the principal executor of apoptosis. Upon activation by upstream signals, it undergoes cleavage to yield active caspase-3 (cleaved caspase-3) [40]. This research elucidated that BQZC could effectively induce apoptosis by upregulating the expression levels of pro-apoptotic proteins, such as cleaved caspase-3 and Bax, while concurrently downregulating the expression of the anti-apoptotic protein Bcl-2. This finding implied that BQZC-induced apoptosis was intricately regulated by the interplay between Bax/Bcl-2 and caspase-3.

In addition, various associations between autophagy and apoptosis have been reported, which hold broad physiological significance. When autophagy becomes excessive, autophagic cells demonstrate a pro-apoptotic action [22]. Moreover, autophagy has been recognized as a vital cellular defense mechanism that enables cells to evade apoptosis-induced demise under specific circumstances, such as pharmacological stimulation [41]. Consequently, it is imperative to elucidate the intricate interplay between the autophagy and apoptosis induced by BQZC in cancer. CQ serves as a widely utilized inhibitor of the autophagic pathway and has been documented to impede autophagy by obstructing the fusion of autophagosomes with lysosomes [42]. Meanwhile, clinical trials have revealed that combining CQ and other antineoplastic drugs can increase sensitivity to chemotherapy or radiotherapy [43]. In our research, the inhibition of autophagy after CQ treatment significantly strengthened the apoptotic effects elicited by BQZC in lung cancer cells. This observation suggests that BQZC-induced autophagy may serve as a stress adaptation mechanism, effectively thwarting cellular apoptosis. These findings indicate that synergistically employing BQZC alongside an autophagy inhibitor could potentiate the anti-lung-cancer efficacy of BQZC.

The AMPK–mTOR pathway is considered to be a major regulator of autophagy [23]. Meanwhile, AMPK, as a sensor of energy molecules, is a positive regulator of autophagy and functions by downregulating mTOR phosphorylation to adapt to energy metabolism. It has been suggested that enhancing AMPK activity may exhibit anti-apoptotic effects on cells [44], and this study demonstrated that BQZC could activate the AMPK–mTOR pathway, which was enhanced through the pre-treatment of tumor cells with Rap, thus significantly increasing the accumulation of autophagosomes and inhibiting apoptosis. Additionally, pre-treatment with ComC before BQZC administration significantly reversed the activation of the induced AMPK–mTOR pathway, leading to a reduction in autophagosomes and the promotion of apoptosis. Moreover, previous RNA-seq and bioinformatics analyses have speculated that ULK1 and BMF are potential downstream targets of AMPK–mTOR for regulating autophagy and apoptosis, respectively. Specifically, ULK1 is the key inducer of autophagosome formation. AMPK can promote autophagy by phosphorylating ULK1 (Ser317 and Ser555) [45,46], while mTOR downregulates autophagy by phosphorylating ULK1 (Ser757) under nutrient-rich conditions [45]. BMF is described as a BH3-only protein belonging to the Bcl-2 protein family and has been reported to function as a pro-apoptotic factor, which initiates cell apoptosis by binding to anti-apoptotic factors (including Bcl-2, Bcl-xL, and Bcl-w), thereby inhibiting malignancies and regulating chemosensitivity [47]. Furthermore, Western blot revealed that, on the one hand, BQZC activates the phosphorylation of mTOR in a dose-dependent manner, increasing the expression level of its downstream effector BMF, thereby promoting cell apoptosis. On the other hand, BQZC treatment induced the phosphorylation of AMPK, directly activating ULK1, an autophagy initiation kinase, thereby promoting cell autophagy and resisting excessive apoptosis. The negative regulation of AMPK on mTOR may be the underlying reason for autophagy inhibiting cell apoptosis. This indicates a significant regulatory relationship between them. These results mechanistically support that BQZC may regulate autophagy, apoptosis, and their interactions on tumors via the AMPK–mTOR–ULK1/BMF signaling pathway to exert anti-lung-cancer effects. Therefore, this study on BMF is of significant importance in exploring the mechanisms of BQZC on autophagy and apoptosis in lung cancer cells. In this study, we elucidated the possible targets of the interaction between autophagy and apoptosis from a new perspective.

Based on the research ideas outlined in this study, purifying effective ingredient combinations from TCM and developing them into innovative Chinese medicine with well-defined pharmacological substances and relatively clear mechanisms of action is one of the significant approaches to innovating new Chinese medicine. Starting from the material foundation, we achieved a coherent model of substance–target–pharmacodynamics–clinical research [48]. The total alkaloids in Mori ramulus are natural water-soluble alkaloids obtained through extraction, separation, and purification. They are an effective component among the many active ingredients in Mori ramulus and were approved for marketing as an original natural hypoglycemic drug in March 2020 [49]. Researchers employed natural medicine research methodologies alongside modern scientific technologies to identify 22 prototype blood components and 8 metabolites present in the plasma of Shexiang Baoxin Pill. Building upon these findings, they elucidated the pharmacological basis and molecular mechanisms underlying the Shexiang Baoxin Pill, thereby establishing a foundation for the future development of innovative traditional Chinese medicine formulations [50]. In light of the findings regarding MCGC and BQZC in this study, it is essential to further optimize the optimal structural ratios of each component to determine their anti-lung-cancer efficacy and examine the processes of their absorption and metabolism within the body. To develop it as a potential innovative TCM, this approach should be patient-centered and driven by clinical value. It is essential to emphasize pharmacokinetic research on effective ingredient combinations, adopt novel dosage forms and technologies to enhance drug absorption and utilization, and strengthen the study of its druggability. This will facilitate the establishment of a solid pharmacological basis for clinical applications, thereby enhancing formulation development and related research efforts.

## 4. Materials and Methods

### 4.1. Materials and Reagents

A total of 26 batches of commercially available CPPP samples from different origins were collected in 4 main production areas, namely, the Guizhou, Yunnan, Sichuan, and Guangxi provinces. They were identified as the dried pseudobulb of *Cremastra appendix* (D. Don) Makino, *Pleione bulbocodioides* (Franch.) Rolfe, and *Pleione yunnanensis* Rolfe by Professor Jinli Shi from the Department of Traditional Chinese Medicine Identification of Beijing University of Chinese Medicine. Their detailed sample information is shown in Table S1.

The standard substances, including gastrodin, dactylorhin A, militarine, batatasin III, 2-(*p*-hydroxybenzyl)-5,3′-dihydroxy-3-methoxybenzyl, blestriarene A, blestriarene B, monbarbatain A, 1-Hydroxybenzyl-2,7-dihydroxy-4-methoxyphenyl, coelonin, malic acid, and citric acid, were purchased from Chengdu Mansite Biotech Co., Ltd. (Chengdu, China). Loroglosin was purchased from Chengdu Purechem-Standard Co., Ltd. (Chengdu, China). The purity of each reference compound was over 98%, as determined using HPLC. MS-grade acetonitrile and methanol were obtained from Fisher Chemical (Waltham, MA, USA). Distilled water was obtained from Watsons Food & Beverage Co., LTD (Guangzhou, China). Furthermore, 95% ethanol was purchased from Beijing Chemical Works (Beijing, China). Fetal bovine serum (FBS) was obtained from the Corning Co., LTD (New York, NY, USA). Roswell Park Memorial Institute (RPMI) 1640, Dulbecco’s Modified Eagle Medium (DMEM) medium, and antibiotics (penicillin and streptomycin) were obtained from Gibco Co., LTD (Gibco-BRL, Grand Island, NY, USA). Dimethyl sulfoxide (DMSO) was obtained from Solarbio Technology Co., Ltd. Beijing, China). Cisplatin (DDP) was obtained from Sigma Co., LTD (Burlington, MA, USA). The CCK-8 kit was obtained from New Cell&Molecular Biotech Co., Ltd. (Suzhuo, China). The Annexin V-FITC Apoptosis Detection Kit was purchased from BD Biosciences Co., LTD (San Jose, CA, USA). CQ, Z-VAD-FMK, Ferrostatin-1 (Fer-1), Dorsomorphin (ComC), and Rapamycin (Rap) were purchased from MedChemExpress (Princeton, NJ, USA). Antibodies against Bcl-2 (26593-1-AP), Bax (50599-2-Ig), LC3B (14600-1-AP), p62 (18420-1-AP), p-mTOR (67778-1-Ig), and p-ULK1 (80218-1-RR) were purchased from Proteintech (Chicago, IL, USA). BMF (#50542) and p-AMPK (#2535) were purchased from Cell Signaling Technology (Beverly, MA, USA). Cleaved-caspase3 (AF7022), HRP-conjugated goat anti-rabbit antibody (S0001), and HRP-conjugated goat anti-mouse antibody (S0002) were purchased from Affinity Biosciences Pty Ltd. (Cincinnati, OH, USA). β-Actin (UM4001) was purchased from Tianjin Youkang Co., Ltd. (Tianjin, China).

### 4.2. Cell Lines and Animals

Human non-small-cell lung cancer A549 and NCI-H1299 cells were purchased from BNCC Technology Co., Ltd. (Beijing, China). The mouse Lewis lung cancer cell line was purchased from Procell Life Science&Technology Co., Ltd. (Wuhan, China). Then, 6–8-weeks-old SPF-grade C57BL/6J male mice weighing 18–22 g were purchased from Beijing Vital River Laboratory Animal Technology Co., Ltd. (animal license number SCXK Beijing 2021-0006). The experimental mice were all raised in the standard barrier environment of the Experimental Animal Department of Beijing University of Traditional Chinese Medicine at 25 ± 2 °C, 55 ± 10% humidity, and a 12 h light/dark cycle. Animal experiments were conducted in accordance with the requirements of International Ethics of Laboratory Animals, with the ethics code BUCM-2023053003-2160.

### 4.3. Preparation of CPPP Extract (CPPPE)

Each batch of CPPP was ground into powder and passed through a 60-mesh sieve. The samples were added with 20 times the volume of 70% ethanol. Then, they were extracted for 90 min via reflux and continuously extracted twice. All filtrates were combined, concentrated into a thick paste, and freeze-dried. The samples of CPPP were obtained and refrigerated in the refrigerator at 4 °C. The extraction rates of the 26 batches of CPPP extract are shown in Table S1.

### 4.4. UHPLC-Q-Exactive Orbitrap MS Fingerprint Analysis

A sample solution of 20 μg·mL^−1^ (calculated as crude drug) was prepared with 60% acetonitrile–water with an appropriate amount of CPPP powder. After ultrasonic treatment and centrifugation, the supernatant was filtered using a 0.22 μm microporous filter membrane, and the filtrate was used as the test solution. A mixed reference solution of about 20 μg·mL^−1^ for each reference was prepared with 60% acetonitrile–water. The above solution was tested under the following conditions.

The CPPP extracts were analyzed using a Thermo UHPLC system connected to a Thermo Q-Exective-Orbitrap-MS system (ThermoScientific, Waltham, MA, USA). Chromatographic separation was performed using a Waters ACQUITY UPLC HSS T3 column (2.1 mm × 100 mm, 1.8 μm). The mobile phase comprised deionized water containing 0.1% formic acid (A) and acetonitrile (B). The gradient elution procedure was as follows: 0–4 min, 2% B; 4–5 min, 2–5% B; 5–20 min, 5–16% B; 20–50 min, 16–45% B; and 50–60 min, 45–80% B. The flow rate was set to 0.3 mL/min. The column temperature was maintained at 30 °C. The injection volume was established at 2 μL. Mass spectra were acquired in a negative mode over a mass range of *m*/*z* 120–1500. The parameters for the ESI source were as follows: the source temperature was set to 400 °C, the ion spray voltage was adjusted to 3.5 kV, and the capillary temperature was maintained at 320 °C. Both the sheath gas and auxiliary gas were nitrogen, sustained at flow rates of 40 arb and 10 arb, respectively. The collision energy gradient utilized values of 20, 40, and 60 eV. The first-level resolution achieved was 70,000, while the secondary resolution reached up to 17,500. All operations mentioned above were conducted using the Xcalibur 4.3 software.

Subsequently, the chromatographic profiles of 26 CPPP samples were imported into The Similarity Evaluation System of Chromatographic Fingerprint for Traditional Chinese Medicine (2012a version), which serves as an instrumental tool for assessing both the similarities and disparities among samples. The MCG-1 and BQZ-1 specimens were designated as the reference maps, with a “time window” width calibrated to 0.5 min. Comprehensive spectrum similarity assessments and comparative fitting analyses were conducted utilizing methods such as average calculations, multi-point corrections, and full-spectrum peak matching. Through meticulous examination of the CPPP fingerprint data, common characteristic peaks were identified for subsequent chemometric analysis.

### 4.5. Cell Culture and Cell Viability Assay

A549 cells were cultured in DMEM supplemented with 10% FBS and antibiotics (including 100 U/mL of penicillin and 100 μg/mL of streptomycin). H1299 cells were maintained in RPMI 1640 medium, containing 10% FBS and antibiotics. Both cell lines were cultured in a 5% CO_2_ humidified incubator at 37 °C.

A Cell Counting Kit-8 (CCK-8) assay was performed to measure the viability of the lung cancer cell lines. Briefly, cells (5000 cells/well) were seeded and cultured to adhere in 96-well plates for 24 h, and then treated with different concentrations of samples for 48 h. The blank group was treated with the medium without the sample and cells, and the control group was treated with the medium (0.1%DMSO) alone. Cisplatin was utilized as the positive control drug. The absorbance was determined at λ = 450 nm using an enzyme-immunoassay instrument (Beijing Create Technology Co., Ltd., Beijing, China). Subsequently, a CCK-8 assay was conducted to determine the cell viability (%), and the viability rate was calculated as (OD_treatment_ − OD_blank_)/(OD_control_ − OD_blank_) × 100%. The half-inhibitory concentration (IC50) of the drug on different cells was calculated using GraphPad prism 5.0.

### 4.6. Cell Migration Assay

For cell migration assays, cells in the logarithmic growth phase were cultured into 96-well plates with 100 μL per well and grown to 80–90% confluence. Then, the cells were scratched using an automatic scratch tool to draw a straight line at the bottom of the 96-well plate, followed by treatment with the appropriate samples containing low-serum culture medium (2% FBS) for 48 h. The 96-well plates were placed in an IncuCyte S3 live cell dynamic imaging system (Sartorius Co., Ltd., Gottingen, Germany) and the scratches were photographed automatically at 0, 12, 24, 36, and 48 h, respectively. Then, then the width of the scratches was statistically analyzed. Cell migration rate (%) = [1 − (scratch distance at 12/24/36/48 h/scratch distance at 0 h)] × 100%.

### 4.7. Cell Apoptosis Analysis

According to the manufacturer’s instructions (BD Bioscience, USA), apoptosis in the cells was assessed using flow cytometry. Tumor cells were seeded into 6-well plates at a density of 5 × 10^5^ cells per well and incubated overnight to allow attachment. Subsequently, the cells underwent the appropriate treatments. To prepare for analysis, the cell samples were digested and collected. Then, 5 µL of Annexin V and 5 µL of FITC were added for staining and incubated for half an hour. Finally, the samples were detected using a flow cytometer (BD LSRFortessa, St. Joseph, CA, USA). Data obtained from the analysis were processed using the FlowJo 10.9.0 software.

### 4.8. AO Staining

The presence of acidic vesicular organelles (AVOs) in cells was determined using acridine orange (AO) staining, with AVOs being indicative of the formation of late-phase autolysosomes involved in autophagic flux. The cells with a fusion rate of 80–90% were seeded into six-well plates at 37 °C for 24 h. The control group and treated group cells were washed twice with PBS, followed by fixation at room temperature using cold 4% paraformaldehyde for 10 min. Subsequently, the cells were stained with AO (5 μg/mL) at room temperature for 15 min. Afterward, they were washed again and examined under a fluorescence microscope (Nikon, Tokyo, Japan).

### 4.9. Establishment of Tumor Xenograft Model

After acclimation for 5 days, LLC cell suspensions (containing 1 × 10^6^ cells) in 0.1 mL of PBS were injected subcutaneously into the armpit of the right foreleg of C57BL/6J mice. Then, lung cancer transplantation models were established separately. When the tumor grew to 50–100 mm^3^, except for the control group, mice with tumors were randomly assigned into 6 groups, with each group containing 10 mice, namely, the model group, positive drug (cisplatin: 6 mg/kg, ip) group, MCG (275.5 mg/kg/d, i.g.) group, MCGC (78.4 mg/kg/d, i.g.) group, BQZ (337.3 mg/kg/d, i.g.) group, and BQZC (130.5 mg/kg/d, i.g.) group (all measured based on the amount of crude medicinal herbs for human use dosage, 12 g·60 kg^−1^). (In the early stage, the research group found that the 70% ethanol extract of CPPP had certain anti-tumor effects at a low dose of 6 g·60 kg^−1^, medium dose of 9·60 kg^−1^, and high dose of 12 g·60 kg^−1^, and inhibited tumor growth in mice in a dose-dependent manner, but its anti-tumor effect was the best at a high dose of 12 g·60 kg^−1^. Therefore, a high dose of 12 g·60 kg^−1^ was selected for the follow-up study in this study. The results are shown in Figure S1). The control group and model group mice were given corresponding volumes of physiological saline by gavage once a day. The groups treated with CPPP (MCG, MCGC, BQZ, and BQZC) were administered with it orally once a day. The cisplatin group was administered via intraperitoneal injection 3 times per week. A total of 2 weeks of medication were administered. Body weight and tumor size were measured every two days after the first drug treatment. The tumor volume calculation formula was as follows: volume (mm^3^) = tumor long diameter (mm) × tumor short diameter (mm)^2^ × 0.5. The blood was collected from the eyeballs of the mice 30 min after the last administration, and when the mice were euthanized, the tumor tissues were completely removed.

### 4.10. Hematoxylin and Eosin (H&E) Staining in Tumor Tissue

After cleansing the surface bloodstains with physiological saline, we immersed the separated tumor tissue in 4% paraformaldehyde fixation solution within a cryopreservation tube, where it remained fixed overnight. The following day, using forceps, we carefully withdrew the fixed tumor tissue from the cryopreservation tube for sectioning, embedding, and deparaffinization. Subsequently, the glass slides were subjected to hematoxylin staining for 2 min and rinsed with distilled water. They were then treated sequentially with 0.1% hydrochloric acid and lithium carbonate and rinsed thoroughly under running water for 15 min. Then, we proceeded to stain the samples with eosin for 1 min. We dehydrated the samples using gradient alcohols and administered a transparent treatment in xylene before sealing the slides with neutral gum. Observations and photographic documentation of the stained specimen were conducted using an inverted optical microscope (Olympus from Tokyo, Japan).

### 4.11. Terminal Deoxynucleotidyl Transferase dUTP Nick-End Labeling (TUNEL) Staining in Tumor Tissue

After fixing the tumor tissue for 24 h, it was washed, dehydrated, embedded in paraffin, and covered with protease K working solution for repair. We followed the instructions of the TUNEL reagent kit and randomly selected the field of view for photography after color development. We observed the apoptosis of tumor tissue cells under an optical microscope and calculated the apoptosis rate (apoptosis rate = number of apoptotic positive cells/total number of cells × 100%).

### 4.12. Immunohistochemistry (IHC)

The embedded paraffin sections were subjected to dewaxing and subsequent antigen retrieval. Specifically, the tumor tissue slices were incubated with 3% hydrogen peroxide to effectively inhibit endogenous peroxidase activities. Following this, a one-hour incubation at 37 °C with diluted goat serum was performed to diminish non-specific staining. Subsequently, the tumor tissues were allowed to interact overnight at 4 °C with the corresponding primary antibody (LC3BII/I, p62, 1:200). On the following day, these samples were incubated with an enzyme-labeled secondary antibody (1:500) along with a chromogenic substrate. This procedure was then followed by hematoxylin counterstaining, dehydration, and embedding. Finally, observations and photographic documentation of the stained slides were conducted using an inverted optical microscope.

### 4.13. Western Blotting Analysis

Cells and tumor tissue samples were lysed using PMSF (Solarbio, Beijing, China). The protein concentrations were determined utilizing an enhanced BCA protein assay kit (Solarbio, Beijing, China), separated via SDS-polyacrylamide gel electrophoresis, and transferred onto a polyvinylidene difluoride (PVDF) membrane (Millipore, Bedford, MA, USA). Following a one-hour incubation for blocking with 5% non-fat milk, the membranes were subjected to two washes with TBST for 15 min each. They were then incubated overnight at 4 °C with a specific primary antibody. After three washes with 1 × TBST solution, the membranes were treated with a secondary antibody at RT for two hours. The detection of protein bands was accomplished utilizing an advanced enhanced chemiluminescence (ECL) system, with images captured using a gel imaging system (Bio-Rad, New York, NY, USA). To serve as a loading control, GAPDH was probed using an anti-GAPDH antibody at a dilution of 1:4000. The identification and quantification of blot density were performed by employing the ImageJ V1.8.0 software.

### 4.14. Obtaining Candidate Active Monomers

Because of the inability to purchase 2-isobutylmalic acid, it was prepared in the laboratory. The hydrolysis of militarine under alkaline conditions was used to obtain 2-isobutyl malic acid. We added 50 mg of militarine and 250 mL of 4% NaOH, and reacted samples at room temperature for 3 h. We adjusted the pH to weakly acidic (pH = 3–5) using HCl in an ice bath to terminate the reaction. Then, we extracted the aqueous phase sequentially with 3/4, 1/2, and 1/4 volumes of ethyl acetate. The ethyl acetate extract was combined three times, concentrated at 55 °C under reduced pressure to a certain volume, and then cooled to room temperature. It was dried with nitrogen blowing to a small volume. After adding a certain volume of water and using ultrasound and 0.22 μM filter membrane filtration, the filtrate was freeze-dried to obtain isobutyl malic acid (light yellow solid, easily hygroscopic and sticky, UPLC-MS purity > 98%). All other candidate active monomers were purchased.

### 4.15. Spectrum–Effect Relationship Analysis

Grey correlation analysis (GRA) assesses the correlation of factors based on their respective development trends. This study employed SPSS 20.0 to calculate the correlations between components and anti-lung-cancer pharmacological indicators, with pharmacological indicators as the parent sequence and peak areas as the subsequence. The R-value served as the correlation coefficient to evaluate the relationships between variables, and a higher R-value indicated a stronger correlation between components and pharmacological indices.

Bivariate correlation analysis (BCA) is a parametric method commonly used to assess the linear relationships between variables. The chromatographic data and anti-lung-cancer pharmacodynamic index data were imported into SPSS 20.0, and then their correlations were examined. We employed the areas of the peaks and pharmacodynamic indicators as the dependent variables. A positive coefficient indicated a positive correlation between components and a comprehensive pharmacological efficacy; conversely, a negative coefficient suggested an inverse relationship. Furthermore, larger absolute values of these coefficients signified stronger correlations among the studied elements.

### 4.16. Serum Drug Chemical Analysis

Blood was extracted from the eyeball and centrifuged at 3000 rpm at 4 °C for 15 min. Serum samples were collected and stored at −80 °C until analysis. Then, a 500 μL serum sample was placed in an Eppendorf (EP) tube an mixed with 1000 μL of acetonitrile and 1000 μL of methanol via vortex mixing. It was centrifuged at 12,000 r/min for 15 min, then the supernatant was taken and blown dry under nitrogen gas. We added 100 μL of 60% acetonitrile to dissolve the residue again. We centrifuged it at 12,000 r/min for 15 min and took the upper material as a sample. The analytical conditions for UHPLC Q-Exactive Orbitrap MS were the same as above.

### 4.17. Statistical Analysis

All in vitro experiments were repeated at least three times and the values are expressed as mean ± SD. For animal experiments, the values are the mean ± SD of 10 mice in each group. GRA and BCA were executed using the SPSS 21.0 software. All statistical data were analyzed using the GraphPad Prism 5.0 and SPSS 21.0 software. Differences were determined using a one-way analysis of variance (ANOVA). Values of *p* < 0.05 were considered to be significant. Significance levels are indicated by * *p* < 0.05, ** *p* < 0.01, and *** *p* < 0.001; ns indicates “not significant”.

## 5. Conclusions

This study found, for the first time, effective ingredient combinations that represent the anti-lung-cancer efficacy of CPPP via the spectrum–effect relationship combined with serum pharmacochemistry, which comprises the following 11 predominant active constituents: monbarbatain A, blestriarene A, blestriarene B, coelonin, 2,7-dihydroxy-1-(4-hydroxybenzyl)-4-methoxyphenanthrene, batatasin III, 2-(*p*-hydroxybenzyl)-3′,5-dihydroxy-3-methoxybibenzyl, gastrodin, 2-isobutylmalic acid, malic acid, and citric acid. Furthermore, 2,7-dihydroxy-1-(4-hydroxybenzyl)-4-methoxyphenanthrene and 2-(*p*-hydroxybenzyl)-3′,5-dihydroxy-3-methoxybibenzyl were first shown to have anti-lung-cancer efficacy. Mechanically, an effective ingredient combination of CPPP induces protective autophagy and apoptosis in lung cancer cells through the AMPK–mTOR–ULK1/BMF signaling pathway. We also identified that BMF is prominent in regulating the interaction between autophagy and cell apoptosis. Collectively, this study elucidated the anti-lung-cancer components of CPPP, and developed effective ingredient combinations that are equivalent to the efficacy of CPPP with clear ingredients and contents, which provides important evidence for the clinical application and deep development of CPPP against lung cancer. Furthermore we revealed the potential mechanism of action underlying this, and also revealed new targets associated with apoptosis and autophagy to some extent, which provides new targets and insights for elucidating the interaction between autophagy and apoptosis in tumor cells.

## Figures and Tables

**Table 1:** MS analysis results of common peaks.

NO.	RT/min	Compounds	Compounds Formula	Theoretical *m*/*z* (Da)	Experimental *m*/*z* (Da)	Mass Error (ppm)	Characteristic Ions	Compounds Type	Source
P1	1.06	2-ethylmalic acid	C_6_H_10_O_5_	161.04554	161.04561	0.43	161.05, 131.03, 113.02, 96.96, 85.03, 73.03, 71.01, 59.01	Organic acids	MCG/BQZ
P2	1.08	D-(+)-glucose	C_6_H_12_O_6_	179.05611	179.05612	0.06	179.05, 113.02, 101.02, 89.02, 71.01, 59.01	Monosaccharide	MCG/BQZ
P3	1.15	sucrose	C_12_H_22_O_11_	341.10893	341.10904	0.32	341.11, 179.06, 161.05, 101.02, 89.02, 71.01, 59.01	Disaccharides	MCG/BQZ
P4	1.33	D-(+)-malic acid	C_4_H_6_O_5_	133.01424	133.01427	0.23	133.01, 115.00, 71.01	Organic acids	MCG/BQZ
P5	1.73	citric acid	C_6_H_8_O_7_	191.01972	191.01982	0.52	191.02, 173.01, 129.02, 111.01, 87.01, 85.03	Organic acids	MCG/BQZ
P6	3.83	gastrodin	C_14_H_20_O_9_	285.10345	285.10376	0.94	123.05, 161.05, 201.03, 265.03, 285.10	Glycosides	MCG/BQZ
P7	8.91	2-isobutyltartaric acid	C_8_H_14_O_6_	205.07176	205.07175	−0.05	205.071, 143.07, 129.06, 115.08, 72.99	Organic acids	MCG/BQZ
P8	11.33	2-isopropylmalic acid	C_7_H_12_O_5_	175.06119	175.06119	0	175.06, 115.04, 113.06, 85.07	Organic acids	MCG/BQZ
P9	13.49	4-benzyl tartrate	C_11_H_12_O_6_	239.05611	239.05627	0.67	239.06, 149.06, 91.06, 72.99	Esters	MCG/BQZ
P10	14.79	eucomic acid	C_11_H_12_O_6_	239.05611	239.05627	0.67	239.06, 179.04, 177.06, 149.06, 107.05	Organic acids	MCG/BQZ
P11	16.09	dactylorhin C	C_14_H_24_O_10_	351.12967	351.13025	1.65	351.13, 171.07, 127.08	Glycosides	MCG/BQZ
P12	17.63	2-isobutylmalic acid	C_8_H_14_O_5_	189.07684	189.07695	0.58	189.08, 129.06, 127.08, 99.08	Organic acids	MCG/BQZ
P13	18.92	coelovirin A	C_21_H_30_O_12_	473.16644	473.16684	0.85	473.17, 159.07, 143.07, 115.08	Glycosides	MCG/BQZ
P14	21.75	2-benzylmalic acid	C_11_H_12_O_5_	223.06119	223.06128	0.40	223.06, 163.04, 161.06, 133.07, 117.07, 91.06	Organic acids	MCG/BQZ
P15	22.53	coelovirin B	C_21_H_30_O_12_	473.16644	473.16684	0.85	473.17, 159.07, 143.07, 115.08	Glycosides	MCG/BQZ
P16	23.61	dactylorhin E	C_27_H_40_O_16_	619.22435	619.22552	1.89	619.23, 439.16, 153.06	Glycosides	MCG/BQZ
P17	25.39	loroglossin	C_34_H_46_O_16_	787.26661	787.26819	2.01	473.17, 115.08	Glycosides	MCG/BQZ
P18	27.23	dactylorhin A	C_40_H_56_O_22_	887.31904	887.32068	1.85	619.22, 439.16, 171.07, 153.06, 127.08	Glycosides	MCG/BQZ
P19	27.29	gymnoside II	C_21_H_30_O_11_	457.17153	457.17215	1.36	427.17, 285.10, 171.07, 153.06, 129.06, 127.08, 123.05, 99.08	Glycosides	MCG/BQZ
P20	27.67	gymnoside	C_21_H_30_O_11_	457.17153	457.17215	1.36	427.17, 285.10, 171.07, 153.06, 129.06, 127.08, 123.05, 99.08	Glycosides	MCG/BQZ
P21	27.75	vandateroside II	C_37_H_44_O_18_	775.24548	775.24701	1.97	507.15, 203.04, 149.06	Glycosides	MCG/BQZ
P22	28.95	cronupapine	C_24_H_28_O_11_	491.15588	491.15649	1.24	491.16, 285.10, 187.04, 161.06, 133.07, 123.05, 91.06	Glycosides	MCG/BQZ
P23	29.64	grammatophylloside A	C_24_H_28_O_11_	491.15588	491.15649	1.24	491.16, 285.10, 187.04, 161.06, 133.07, 123.05, 91.06	Glycosides	MCG/BQZ
P24	31.57	militarine	C_34_H_46_O_17_	725.2717	725.27332	2.10	457.17, 153.06, 129.06, 127.08, 123.05, 99.08	Glycosides	MCG/BQZ
P25	32.51	gymnoside III	C_42_H_58_O_23_	929.32961	929.33179	2.35	929.31, 661.24, 473.17, 143.07, 129.06, 123.04, 115.08, 99.08	Glycosides	MCG/BQZ
P26	32.68	grammatophylloside B	C_37_H_44_O_17_	760.25605	760.25757	1.89	491.16, 187.04, 161.06, 123.05, 91.06	Glycosides	MCG/BQZ
P27	34.11	shancigusin H	C_49_H_62_O_24_	1033.35582	1033.35803	2.14	1033.36, 765.26, 619.23, 439.16, 187.04, 153.06, 145.03, 119.05	Glycosides	MCG/BQZ
P28	34.24	gymnoside V	C_49_H_62_O_23_	1019.36639	1019.36877	2.24	749.26, 569.20, 439.16, 153.06	Glycosides	MCG/BQZ
P29	36	gymnoside VI	C_49_H_62_O_23_	1019.36639	1019.36877	2.24	1063.35, 883.30, 795.27, 439.16, 175.04, 153.06	Glycosides	MCG/BQZ
P30	39.1	gymnoside IV	C_49_H_62_O_23_	1019.36639	1019.36877	2.24	749.26, 439.16, 153.06	Glycosides	MCG/BQZ
P31	39.53	gymnoside V isomer	C_49_H_62_O_23_	1019.36639	1019.36877	2.24	431.13504, 145.02942, 99.08143	Glycosides	MCG/BQZ
P32	40.18	Unknown1	C_43_H_52_O_19_	871.30301	871.30463	1.87	871.29559, 603.20844, 543.18555, 457.17328, 431.1347, 307.082, 145.02956, 129.05573, 123.04508, 117.03455, 99.08147	—	MCG/BQZ
P33	40.33	Unknown2	C_31_H_38_O_14_	633.21887	633.22015	2.02	461.14569, 377.09384, 193.05049, 175.03984, 160.01656, 127.07623, 99.08152	—	MCG/BQZ
P34	41.53	batatasin III	C_15_H_16_O_3_	243.10267	243.10284	0.90	243.10, 227.07, 174.96, 158.98, 146.96, 130.98	Phenanthrenes	MCG/BQZ
P35	44.07	2,7-dihydroxy-1-(4- hydroxybenzyl)-4- methoxyphenanthrene	C_22_H_18_O_4_	345.11323	345.11353	0.87	345.11, 330.09, 302.09, 237.06	Phenanthrenes	MCG/BQZ
P36	44.35	shancilin	C_30_H_28_O_6_	483.18131	483.18192	1.26	483.18, 467.15, 347.09, 332.07	Phenanthrenes	MCG/BQZ
P37	45.48	2,7-dihydroxy-4- methoxy-phenanthrene	C_15_H_12_O_3_	255.06628	255.06656	1.10	255.06656, 240.04318, 208.97038, 180.97562, 164.98051, 136.98523, 120.990094, 75.00868	Phenanthrenes	MCG/BQZ
P38	45.66	blestriarene A	C_30_H_26_O_6_	481.16566	481.1662	1.12	481.17, 465.13, 224.05	Phenanthrenes	MCG/BQZ
P39	46.22	2-(*p*-hydroxybenzyl)-3′,5-dihydroxy-3-methoxybibenzyl	C_22_H_22_O_4_	349.14453	349.14487	0.97	349.14, 255.10, 240.08, 93.03	Bibenzyl	MCG/BQZ
P40	46.59	blestriarene B	C_30_H_24_O_6_	479.15001	479.15067	1.38	479.15, 464.13, 421.11, 379.10, 224.05	Phenanthrenes	MCG/BQZ
P41	47.19	monbarbatain A	C_30_H_22_O_6_	477.13436	477.13501	1.36	477.13, 462.11, 447.09, 430.08, 419.09, 391.10	Phenanthrenes	MCG/BQZ
P42	48.42	coelonin	C_15_H_14_O_3_	241.08701	241.08743	1.74	241.08743, 226.06355, 198.06837, 172.94029, 118.96604, 86.40402, 69.23640	Phenanthrenes	MCG/BQZ
P43	48.48	bulbocodin C	C_29_H_28_O_5_	501.19187	501.19244	1.14	455.19, 361.15, 93.03	Bibenzyl	MCG/BQZ

—: no data.

**Table 2:** Active ingredients screening based on grey correlation analysis and bivariate analysis.

GRA (Top Ten) ∩ BCA (Top Ten)	The Screened Active Ingredients (Peaks)			
	A549 Proliferation Inhibition	A549 Migration Inhibition	H1299 Proliferation Inhibition	H1299 Migration Inhibition
MCG	P6, P12, P15, P20, P34	P34, P35 P40, P41, P42	P6, P12, P15, P22, P25, P26, P34	P12, P21, P34, P42,
	P6, P12, P15, P20, P21, P22, P25, P26, P34, P35, P40, P41, P42			
BQZ	P5, P6, P24, P34, P35, P38, P39	P12, P18, P24, P34, P35, P38, P39	P6, P7, P24, P34, P38	P4, P29, P38
	P4, P5, P6, P7, P12, P18, P24, P29, P34, P35, P38, P39			

GRA: grey relation analysis; BCA: bivariate correlation analysis.

**Table 3:** Identification of prototype components in mice serum after oral administration of MCG/BQZ by UPLC-Q-Exactive Orbitrap MS.

NO.	RT/min	Adducts	Compounds	Compounds Formula	Theoretical *m*/*z* (Da)	Mass Error (ppm)	Characteristic Ions	Source
P1	1.09	[M − H]^−^	isocitric acid	C_6_H_8_O_7_	191.01971	−0.05	111.01, 87.01, 85.03, 191.02, 133.05	MCG/BQZ
P2	1.23	[M − H]^−^	malic acid	C_4_H_6_O_5_	133.01418	−0.45	115.00, 71.01, 133.01	MCG/BQZ
P3	1.33	[M − H]^−^	2-methylmalic acid	C_5_H_8_O_5_	147.02995	0.41	147.03, 129.02, 115.00, 87.01, 85.03	MCG/BQZ
P4	1.42	[M − H]^−^	methylmalonic acid	C_4_H_6_O_4_	117.01929	−0.34	73.03, 117.02	MCG/BQZ
P5	1.56	[M − H]^−^	2-ethylmalic acid	C_6_H_10_O_5_	161.04549	−0.31	57.03, 99.05, 73.03, 73.03, 131.03	MCG/BQZ
P6	1.64	[M − H]^−^	citric acid	C_6_H_8_O_7_	191.01967	−0.26	111.01, 87.01, 85.03, 191.02, 133.05	MCG/BQZ
P7	3.27	[M − H]^−^	*o*-hydroxybenzoic acid	C_7_H_6_O_3_	137.02428	−0.95	108.02, 93.03, 80.03	BQZ
P8	4.11	[M + HCOO]^−^	gastrodin	C_13_H_18_O_7_	331.10364	0.57	123.05, 161.05, 285.10, 331.10	MCG/BQZ
P9	5.7	[M − H]^−^	*M − H*ydroxybenzoic acid	C_7_H_6_O_3_	137.02432	−0.66	108.02, 93.03, 80.03	BQZ
P10	7.11	[M − H]^−^	2-isobutyltartaric acid	C_8_H_14_O_6_	205.07161	−0.73	115.08, 205.07, 129.06, 143.07, 72.99	MCG/BQZ
P11	9.66	[M − H]^−^	2-isopropylmalic acid	C_7_H_12_O_5_	175.06114	−0.29	115.04, 175.06, 146.96, 113.06, 85.07	MCG/BQZ
P12	12.46	[M − H]^−^	eucomic acid	C_11_H_12_O_6_	239.05623	0.50	239.06, 179.03, 177.06, 133.07	MCG/BQZ
P13	12.95	[M − H]^−^	dactylorhin C	C_14_H_24_O_10_	351.12976	0.26	127.08, 171.07, 351.13	MCG/BQZ
P14	13.51	[M − H]^−^	2-isobutylmalic acid	C_8_H_14_O_5_	189.07695	0.58	129.06, 189.08, 127.08, 99.08	MCG/BQZ
P15	13.9	[M − H]^−^	dactylorhin E	C_27_H_40_O_16_	619.22552	1.89	153.06, 439.16, 123.05	BQZ
P16	13.94	[M − H]^−^	coelovirin B	C_21_H_30_O_12_	473.16638	−0.13	115.08, 159.07, 143.07	MCG
P17	14.02	[M − H]^−^	*p*-hydroxycinnamic acid	C_9_H_8_O_3_	163.04010	0.25	119.05, 163.04	MCG/BQZ
P18	14.36	[M − H]^−^	2-benzylmalic acid	C_11_H_12_O_5_	223.06119	0.00	223.06, 163.04, 161.06, 133.06	MCG/BQZ
P19	14.72	[M − H]^−^	gymnoside	C_21_H_30_O_11_	457.17194	0.90	123.05, 129.06, 127.08, 153.06, 285.10	MCG/BQZ
P20	15.02	[M − H]^−^	grammatophylloside A	C_24_H_28_O_11_	491.15628	0.81	133.07, 123.05, 161.06, 187.04, 285.10	MCG/BQZ
P21	15.02	[M + HCOO]^−^	militarine	C_34_H_46_O_17_	771.27332	2.10	457.17, 123.05, 127.08, 129.06, 153.06	MCG/BQZ

**Table 4:** Identification of metabolites components in mice serum after oral administration of MCG/BQZ by UPLC-Q-Exactive Orbitrap MS.

NO.	RT/min	Adducts	Compounds	Compounds Formula	Theoretical *m*/*z* (Da)	Mass Error (ppm)	Characteristic Ions	Source
M1	4.11	[M + HCOO]^−^	gastrodin	C_13_H_18_O_7_	331.10364	0.57	123.05, 161.05, 285.10, 331.10	MCG/BQZ
M2	7.11	[M − H]^−^	2-isobutyltartaric acid	C_8_H_14_O_6_	205.07161	−0.73	115.08, 205.07, 129.06, 143.07, 72.99	MCG/BQZ
M3	12.46	[M − H]^−^	eucomic acid	C_11_H_12_O_6_	239.05623	0.50	239.06, 179.03, 177.06, 133.07	MCG/BQZ
M4	13.12	[M − H]^−^	*p*-hydroxybenzaldehyde	C_7_H_6_O_2_	121.02953	0.25	121.03, 108.02	MCG/BQZ
M5	13.51	[M − H]^−^	2-isobutylmalic acid	C_8_H_14_O_5_	189.07695	0.58	129.06, 189.08, 127.08, 99.08	MCG/BQZ
M6	14.36	[M − H]^−^	2-benzylmalic acid	C_11_H_12_O_5_	223.06119	0.00	223.06, 163.04, 161.06, 133.06	MCG/BQZ
M7	14.48	[M + C_12_H_15_O_12_]^−^	blestriarene A	C_30_H_26_O_6_	833.23035	0.62	113.02, 657.20, 466.14, 175.02, 833.23	MCG/BQZ
M8	14.54	[M + C_12_H_15_O_12_]^−^	1-*p*-hydroxybenzyl-4,7-dihydroxy-2-methoxy-9,10-dihydrophenanthrene	C_22_H_20_O_4_	699.19275	−0.43	523.16, 113.02, 253.09, 175.02, 347.13	MCG/BQZ
M9	14.63	[M + C_12_H_15_O_12_]^−^	2,7-dihydroxy-1-(4- hydroxybenzyl)-4- methoxyphenanthrene	C_22_H_18_O_4_	697.17792	0.75	521.15, 113.02, 175.02, 251.07, 345.11	MCG/BQZ
M10	14.63	[M + C_12_H_15_O_12_]^−^	blestriarene B	C_30_H_24_O_6_	831.21423	0.06	113.02, 655.18, 479.15, 175.02	MCG/BQZ
M11	14.74	[M + C_12_H_15_O_12_]^−^	monbarbatain A	C_30_H_22_O_6_	829.19922	0.83	113.02, 653.17, 477.14, 175.02	MCG/BQZ
M12	14.76	[M + C_12_H_15_O_12_]^−^	bulbocodioidins G	C_31_H_24_O_7_	859.20831	−0.92	113.02, 683.18, 507.15, 175.02	MCG
M13	14.79	[M + C_6_H_7_O_6_]^−^	coelonin	C_15_H_14_O_3_	417.11938	0.67	113.02, 85.03, 121.03, 175.02, 241.09	MCG/BQZ
M14	14.81	[M + C_12_H_15_O_12_]^−^	2-(*p*-hydroxybenzyl)-3′,5-dihydroxy-3-methoxybibenzyl	C_22_H_22_O_4_	701.20776	−1.34	525.18, 113.02, 255.10, 349.15	MCG/BQZ
M15	15.2	[M − H]^−^	*p*-hydroxybenzoic acid	C_7_H_6_O_3_	137.02429	−0.88	93.03, 137.02, 108.02	MCG/BQZ
M16	15.2	[M + C_6_H_7_O_6_]^−^	2,7-dihydroxy-4- methoxy-phenanthrene	C_15_H_12_O_3_	415.10377	0.77	415.10, 239.07, 224.05, 175.03, 113.02	MCG
M17	15.47	[M + C_6_H_7_O_6_]^−^	batatasin III	C_15_H_16_O_3_	419.13501	0.62	113.02, 243.10, 85.03, 227.07	MCG/BQZ

## Data Availability

All the data have appeared in the paper. Data will be available upon request to the corresponding authors.

## Supplementary Materials

The following supporting information can be downloaded at: https://www.mdpi.com/article/10.3390/ph17111515/s1, Figure S1: The effect of different doses of CPPP on the growth of LLC xenografted tumor in vivo. (A) Tumor images. (B) The body weight was measured every three days. (C) Tumor growth curves. (D) Tumor weight. (E) Tumor histopathological morphology (HE staining, ×200, *n* = 3), a—Model group, b—Positive drug group, c—CPPP-L group, d—CPPP-M group, amd e—CPPP-H group, Note: The red arrow indicates mitotic phase, the black arrow indicates necrotic cells, and the yellow arrow indicates infiltration of inflammatory cells. (F) Organ index (Liver index, Renal index, Spleen index, Thymus index). Data are presented as mean ± SD, * *p* < 0.05, ** *p* < 0.01, *** *p* < 0.001 vs. model group; ### *p* < 0.001 vs. control group; ns, not significant; Figure S2: The effect of MCG-1 on the proliferation ability of A549 and H1299 cells. A549 (A) and H1299 (B) cells were treated with sample MCG-1 of various concentrations for 24 h and 48 h, and CCK-8 assay was performed to detect the viability of these cell lines. Data are mean ± SD, *n* = 6, * *p <* 0.05, ** *p* < 0.01, *** *p* < 0.001, Treatment group vs. Control; Figure S3: Possible metabolic pathways of militarine (P21); Figure S4: The effect of 11 candidate monomer components on A549 and H1299 cells viability and migration rate. (A) monbarbatain A, (B) blestriarene A, (C) blestriarene B, (D) 2,7-dihydroxy-1-(4-hydroxybenzyl)-4-methoxyphenanthrene, (E) coelonin, (F) batatasin III, (G) 2-(*p*-hydroxybenzyl)-3′,5-dihydroxy-3-methoxybibenzyl, (H) gastrodin, (I) 2-isobutylmalic acid, (G) malic acid, (K) citric acid. Data are mean ± SD, *n* = 6, * *p* < 0.05, ** *p* < 0.01, *** *p* < 0.001, Treatment group vs. Control; Figure S5: The effect of positive drugs on the proliferation ability of A549 and H1299 cells. A549 (A), H1299 (B) were treated with cisplatin of various concentrations for 48 h, and CCK-8 assay was performed to detect the viability of these cell lines. Data are mean ± SD, *n* = 6, * *p <* 0.05, ** *p* < 0.01, *** *p* < 0.001, Treatment group vs. Control; Figure S6: The effect of positive drugs on the migration ability of A549 and H1299 cells. A549 (A), H1299 (B) were treated with cisplatin of various concentrations for 48 h, and wound healing assay was conducted to determine the migrative ability of these cell lines. Data are mean ± SD, *n* = 6, * *p <* 0.05, ** *p* < 0.01, *** *p* < 0.001, Treatment group vs. Control; Table S1: Source information and yield of 26 batches of CPPP samples; Table S2: Results of UHPLC-MS fingerprint of precision, repeatability, and stability (*n* = 6); Table S3: Results of similarity analysis of 12 MCG samples and 14 BQZ samples; Table S4: Results of proliferative vitality efficacy indexes determination of 12 batches of MCG and 14 batches of BQZ; Table S5: Correlations and grade of GRA analysis of MCG; Table S6: Correlations and grade of GRA analysis of BQZ; Table S7: Correlations and grade of BCA analysis of MCG; Table S8: Correlations and grade of BCA analysis of BQZ; Table S9: The content and proportion of each monomer in MCGC; Table S10: The content and proportion of each monomer in BQZC; Table S11: Comparison of tumor inhibition rates among different groups.

## Abbreviations

AMPK, AMP-activated protein kinase; AO, acridine orange; AVOs, acidic vesicular organelles; BCA, bivariate correlation analysis; Bax, Bcl-2 associated X; Bcl-2, B cell lymphoma 2; BQZC, anti-lung-cancer effective ingredients combination of BQZ; CCK-8, Cell Counting Kit-8; CQ, chloroquine; DMSO, dimethyl sulfoxide; FITC, fluorescein isothiocyanate; GRA, Grey relational analysis; HE, hematoxylin–eosin staining; IHC, immunohistochemical; LC3, microtubule-associated protein 1A/1B-light chain 3; MCGC, anti-lung-cancer effective ingredients combination of MCG; mTOR, mammailan target of rapamycin; PARP, cleaved-poly (ADP-ribose) polymerase; PMSF, phenyl methane sulfonyl fluoride; PI, Annexin V-propidium iodide; TUNEL, terminal deoxynucleotidyl transferase dUTP Nick-end labeling; UHPLC, ultra-high-performance liquid chromatography; ULK1, unc-51 like autophagy activating kinase 1.
