# Supplementary material for: Cremastrae Pseudobulbus Pleiones Pseudobulbus (CPPP) Against Non-Small-Cell Lung Cancer: Elucidating Effective Ingredients and Mechanism of Action
Source: Pharmaceuticals (Basel). 2024 Nov 11;17(11):1515. doi: 10.3390/ph17111515 (PMC11597303; doi:10.3390/ph17111515)
Supplement: Supplementary file 1 [file pharmaceuticals-17-01515-s001.zip › pharmaceuticals-3282693-supplementary.pdf]

Supplementary Materials

Figure S1. The effect of different doses of CPPP on the growth of LLC xenografted tumor in vivo. (A) Tumor images. (B) The body weight was measured every three days. (C) Tumor growth curves. (D) Tumor weight. (E) Tumor histopathological morp hology (HE staining, ×200, *n*=3), a-Model group b-Positive drug group c-CPPP-L group d-CPPP-M group e-CPPP-H group, Note: The red arrow indicates mitotic phase, the black arrow indicates necrotic cells, and the yellow arrow indicates infiltration of inflammatory cells. (F) Organ index (Liver index, Renal index, Spleen index, Thymus index). Data are presented as mean±SD, $^{*}p$<0.05, $^{**}p$<0.01, $^{***}p$<0.001 vs. model group; $^{\#\#\#}p$<0.001 vs. control group; ns, not significant.

Figure S2. The effect of MCG-1 on the proliferation ability of A549 and H1299 cells. A549 (A) and H1299 (B) cells were treated with sample MCG-1 of various concentrations for 24h and 48h, and CCK-8 assay was performed to detect the viability of these cell lines. Data are mean ± SD, *n*=6, $^{*}P$<0.05, $^{**}P$<0.01, $^{***}P$<0.001, Treatment group vs. Control.

Figure S3. Possible metabolic pathways of militarine (P21)

Figure S4. The effect of 11 candidate monomer components on A549 and H1299 cells viability and migration rate. (A) monbarbatain A, (B) blestriarene A, (C) blestriarene B, (D) 2,7-dihydroxy-1-(4-hydroxybenzyl)-4-methoxyphenanthrene, (E) coelonin, (F) batatasin III, (G) 2-(p-hydroxybenzyl)-3’,5-dihydroxy-3-methoxybibenzyl, (H) gastrodin, (I) 2-isobutylmalic acid, (G) malic acid, (K) citric acid. Data are mean±SD, *n*=6, **P*<0.05, ***P*<0.01, ****P*<0.001, Treatment group vs. Control.

Figure S5. The effect of positive drugs on the proliferation ability of A549 and H1299 cells. A549 (A), H1299 (B) were treated with cisplatin of various concentrations for 48h, and CCK-8 assay was performed to detect the viability of these cell lines. Data are mean±SD, *n*=6, $^{*}P$<0.05, $^{**}P$<0.01, $^{***}P$<0.001, Treatment group vs. Control.

Figure S6. The effect of positive drugs on the migration ability of A549 and H1299 cells. A549 (A), H1299 (B) were treated with cisplatin of various concentrations for 48h, and wound healing assay was conducted to determine the migrative ability of these cell lines. Data are mean±SD, *n*=6, $^{*}P$<0.05, $^{**}P$<0.01, $^{***}P$<0.001, Treatment group vs. Control.

Table S1. Source information and yield of 26 batches of CPPP samples.

Table S2 Results of UHPLC-MS fingerprint of precision, repeatability, and stability (*n*=6).

Table S3. Results of similarity analysis of 12 MCG samples and 14 BQZ samples.

Table S4 Results of proliferative vitality efficacy indexes determination of 12 batches of MCG and 14 batches of BQZ.

Table S5. Correlations and grade of GRA analysis of MCG.

Table S6. Correlations and grade of GRA analysis of BQZ.

Table S7. Correlations and grade of BCA analysis of MCG.

Table S8. Correlations and grade of BCA analysis of BQZ

Table S9. The content and proportion of each monomer in MCGC.

Table S10. The content and proportion of each monomer in BQZC.

Table S11. Comparison of tumor inhibition rates among different groups.

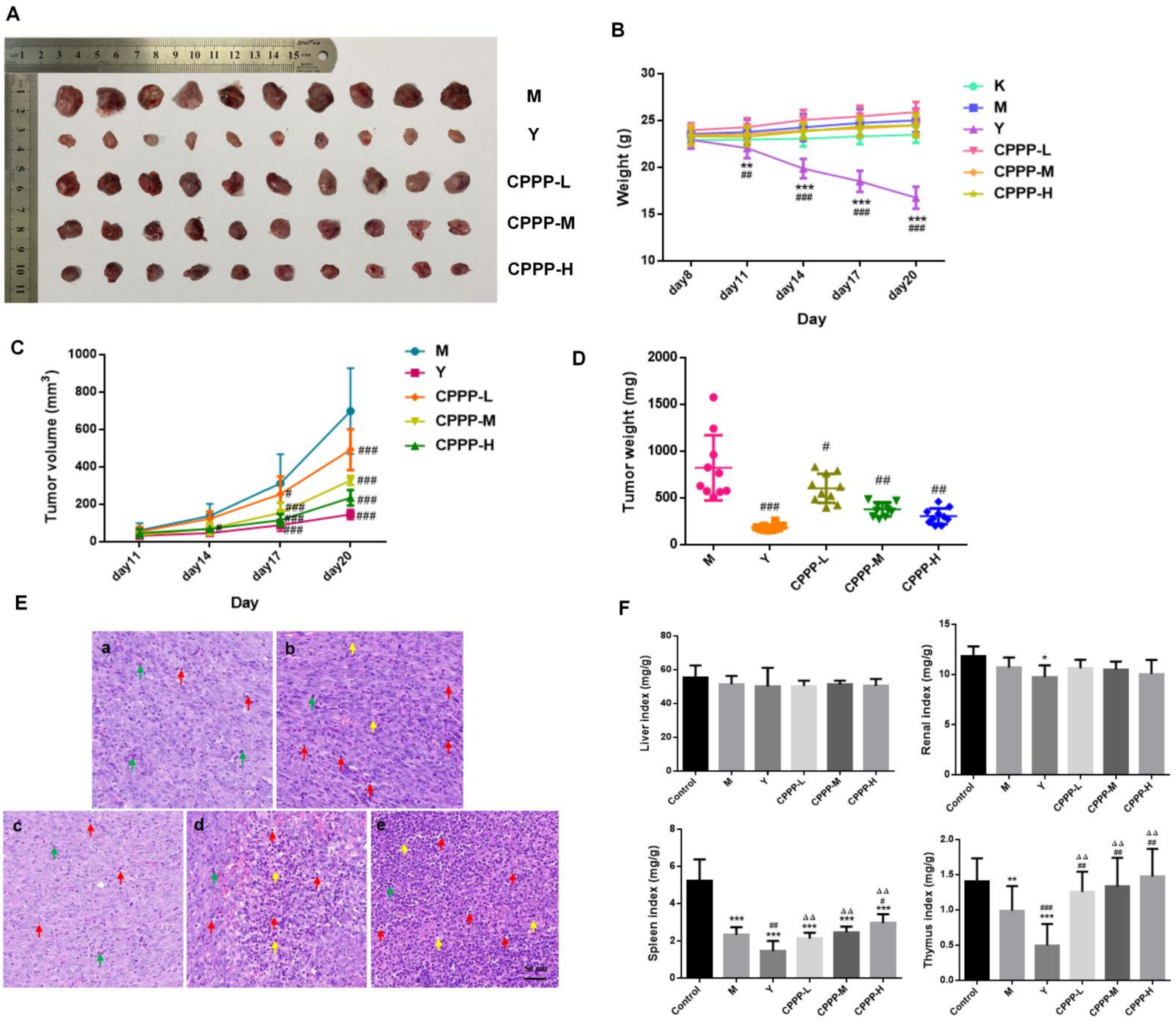

Figure S1. The effect of different doses of CPPP on the growth of LLC xenografted tumor in vivo. (A) Tumor images. (B) The body weight was measured every three days. (C) Tumor growth curves. (D) Tumor weight. (E) Tumor histopathological morp hology (HE staining, ×200, *n*=3), a-Model group b-Positive drug group c-CPPP-L group d-CPPP-M group e-CPPP-H group, Note: The red arrow indicates mitotic phase, the black arrow indicates necrotic cells, and the yellow arrow indicates infiltration of inflammatory cells. (F) Organ index (Liver index, Renal index, Spleen index, Thymus index). Data are presented as mean±SD, $^{*}p$<0.05, $^{**}p$<0.01, $^{***}p$<0.001 vs. model group; $^{###}p$<0.001 vs. control group; ns, not significant.

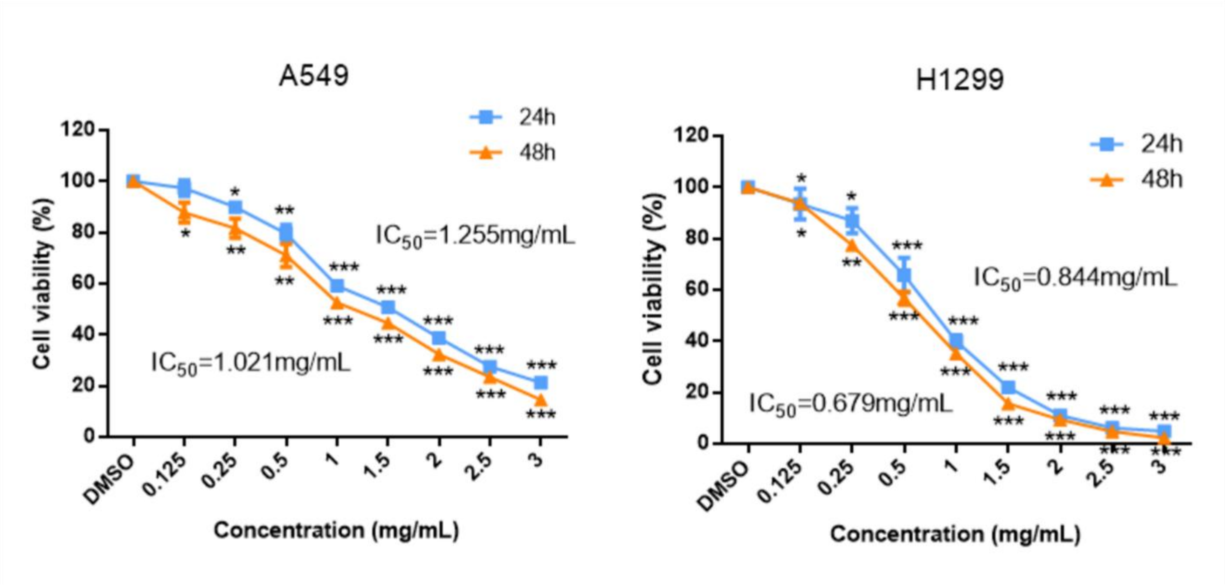

Figure S2. The effect of MCG-1 on the proliferation ability of A549 and H1299 cells. A549 (A) and H1299 (B) cells were treated with sample MCG-1 of various concentrations for 24h and 48h, and CCK-8 assay was performed to detect the viability of these cell lines. Data are mean ± SD, n=6, *P<0.05, **P<0.01, ***P<0.001, Treatment group vs. Control.

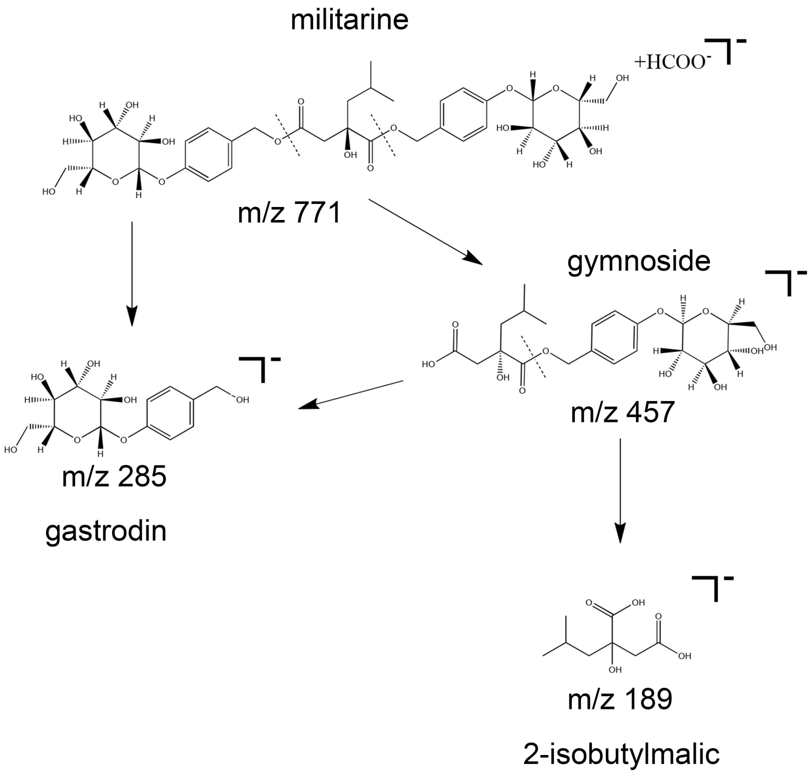

Figure S3. Possible metabolic pathways of militarine (P21)

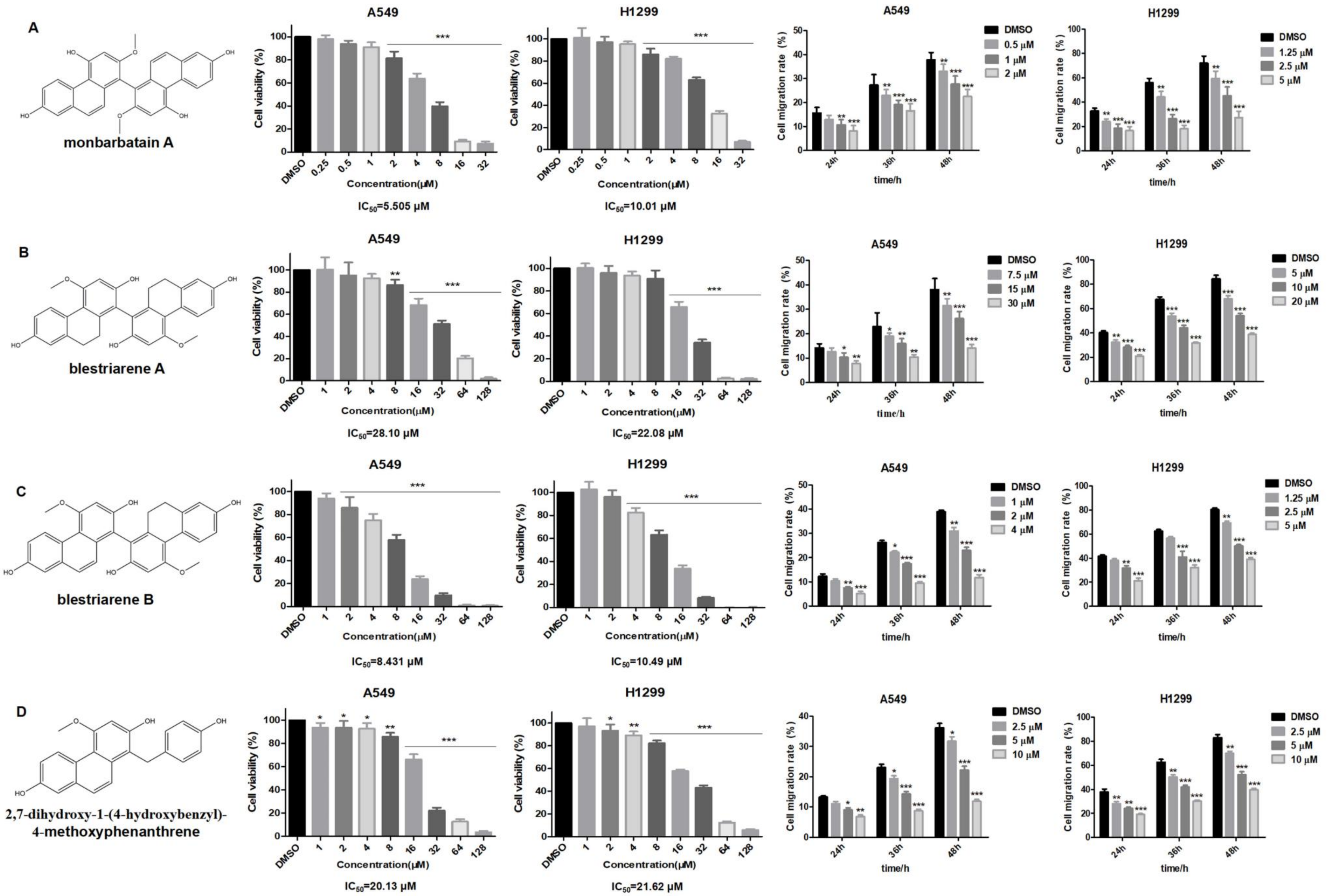

A
monbarbatain A
A549
Cell viability (%)
Concentration(μM)
IC50=5.505 μM
H1299
IC50=10.01 μM
A549
Cell migration rate (%)
time/h
DMSO
0.5 μM
1 μM
2 μM
H1299
DMSO
1.25 μM
2.5 μM
5 μM
B
blestriarene A
A549
IC50=28.10 μM
H1299
IC50=22.08 μM
DMSO
7.5 μM
15 μM
30 μM
DMSO
5 μM
10 μM
20 μM
C
blestriarene B
A549
IC50=8.431 μM
H1299
IC50=10.49 μM
DMSO
1 μM
2 μM
4 μM
DMSO
1.25 μM
2.5 μM
5 μM
D
2,7-dihydroxy-1-(4-hydroxybenzyl)-4-methoxyphenanthrene
A549
IC50=20.13 μM
H1299
IC50=21.62 μM
DMSO
2.5 μM
5 μM
10 μM
DMSO
2.5 μM
5 μM
10 μM

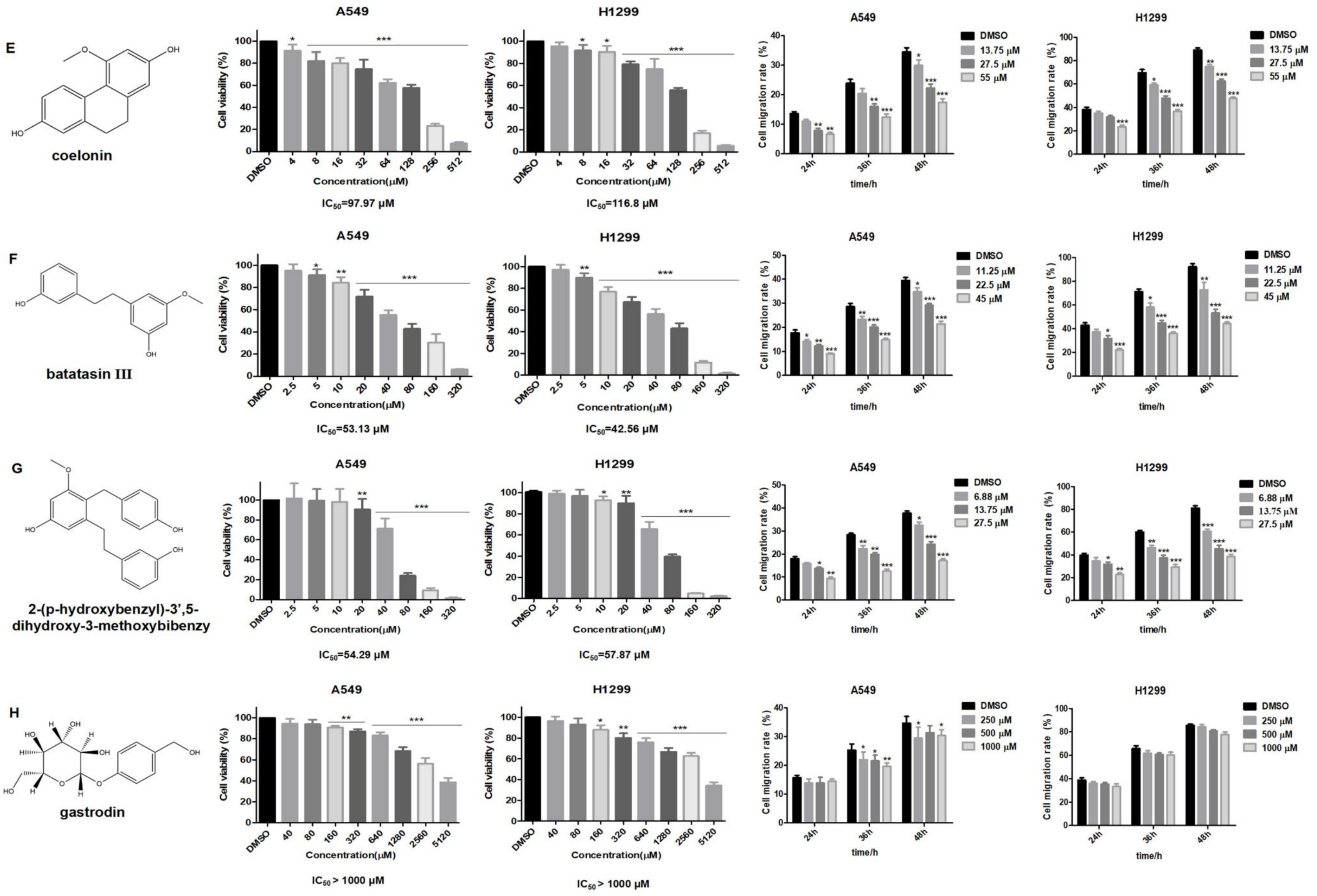
E
coelonin
A549
Cell viability (%)
Concentration(μM)
DMSO
4
8
16
32
64
128
256
512
IC50=97.97 μM
H1299
IC50=116.8 μM
Cell migration rate (%)
time/h
24h
36h
48h
DMSO
13.75 μM
27.5 μM
55 μM
F
batatasin III
2.5
5
10
20
40
80
160
320
IC50=53.13 μM
IC50=42.56 μM
11.25 μM
22.5 μM
45 μM
G
2-(p-hydroxybenzyl)-3',5-dihydroxy-3-methoxybibenzy
IC50=54.29 μM
IC50=57.87 μM
6.88 μM
13.75 μM
27.5 μM
H
gastrodin
40
80
160
320
640
1280
2560
5120
IC50 > 1000 μM
IC50 > 1000 μM
250 μM
500 μM
1000 μM

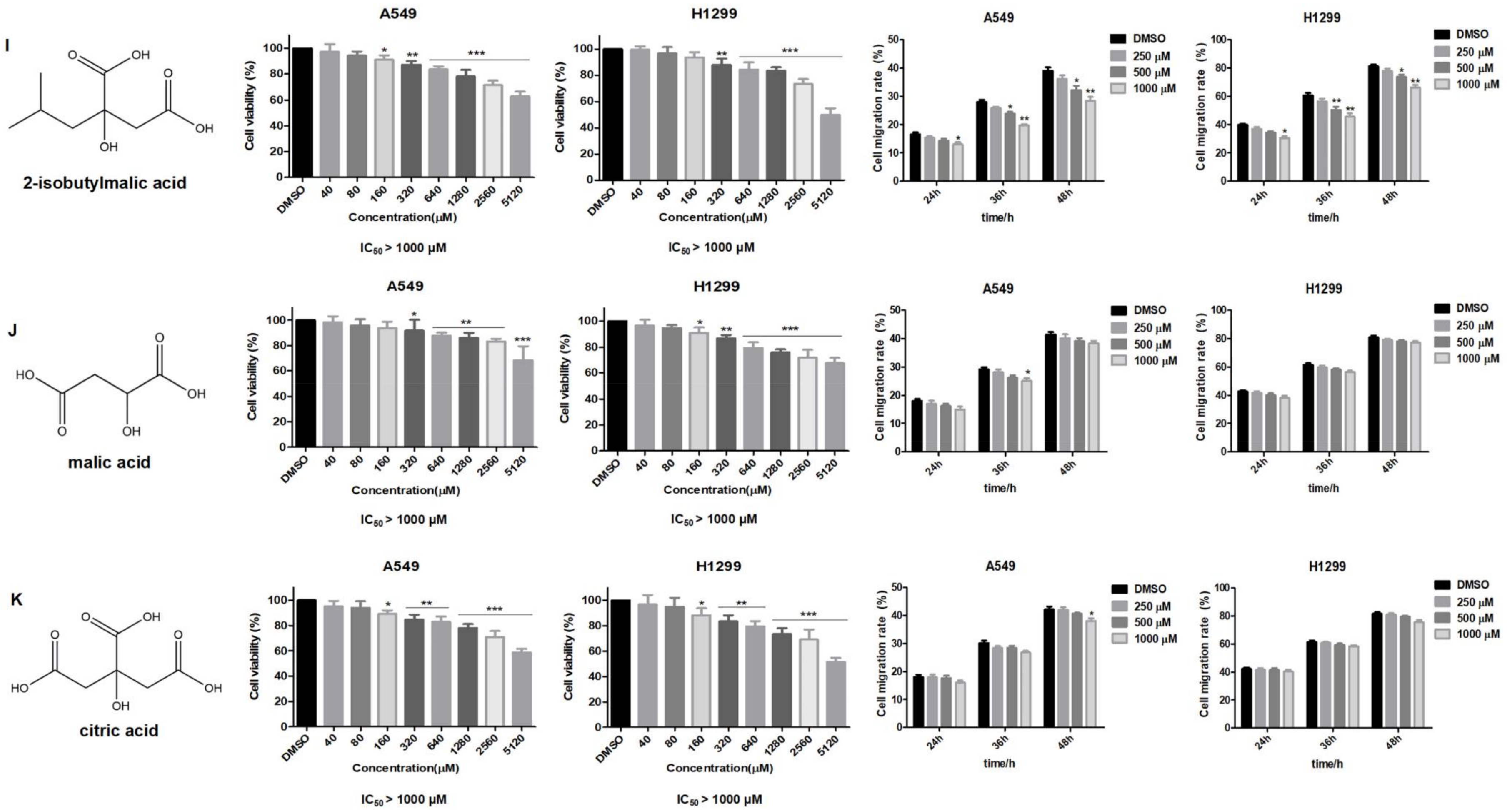

Figure S4. The effect of 11 candidate monomer components on A549 and H1299 cells viability and migration rate. (A) monbarbatain A, (B) blestriarene A, (C) blestriarene B, (D) 2,7-dihydroxy-1-(4-hydroxybenzyl)-4-methoxyphenanthrene, (E) coelonin, (F) batatasin III, (G) 2-(p-hydroxybenzyl)-3',5-dihydroxy-3-methoxybibenzyl, (H) gastrodin, (I) 2-isobutylmalic acid, (G) malic acid, (K) citric acid. Data are mean±SD, n=6, *P<0.05, **P<0.01, ***P<0.001, Treatment group vs. Control.

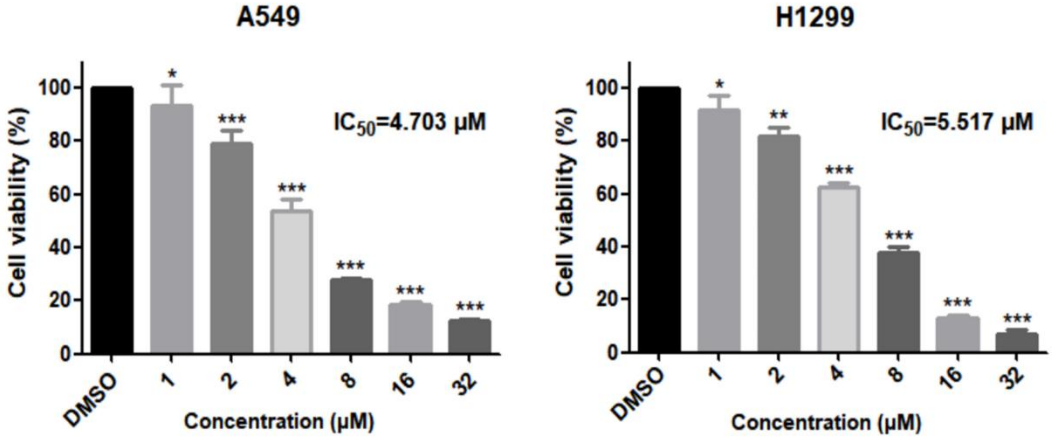

Figure S5. The effect of positive drugs on the proliferation ability of A549 and H1299 cells. A549 and H1299 cells were treated with cisplatin of various concentrations for 48h, and CCK-8 assay was performed to detect the viability of these cell lines. Data are mean±SD, n=6, $^{*}P$<0.05, $^{**}P$<0.01, $^{***}P$<0.001, Treatment group vs. Control.

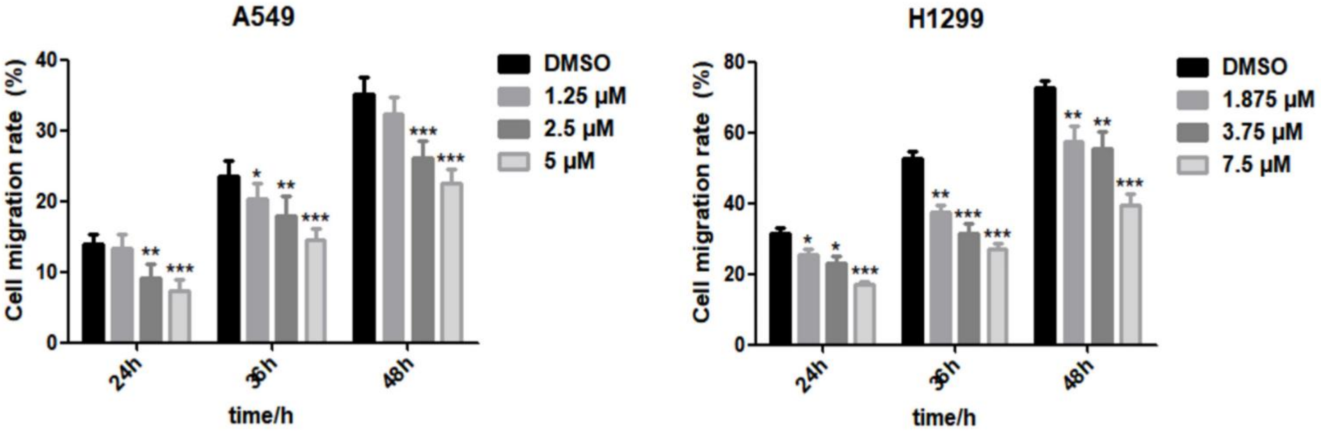

Figure S6. The effect of positive drugs on the migration ability of A549 and H1299 cells. A549 and H1299 were treated with cisplatin of various concentrations for 48h, and wound healing assay was conducted to determine the migrative ability of these cell lines. Data are mean±SD, n=6, $^{*}P$<0.05, $^{**}P$<0.01, $^{***}P$<0.001, Treatment group vs. Control.

Table S1. Source information and yield of 26 batches of CPPP samples.

NO.	Product specifications	Region	Plant origin	Yield（%）
MCG-1	Maocigu	Guizhou, Zunyi	*Cremastra appendix* (D. Don) Makino	9.29%
MCG-2	Maocigu	Guizhou, Zunyi	*Cremastra appendix* (D. Don) Makino	9.76%
MCG-3	Maocigu	Guizhou, Zunyi	*Cremastra appendix* (D. Don) Makino	9.29%
MCG-4	Maocigu	Guangxi, Yulin	*Cremastra appendix* (D. Don) Makino	6.50%
MCG-5	Maocigu	Guangxi, Yulin	*Cremastra appendix* (D. Don) Makino	7.62%
MCG-6	Maocigu	Guangxi, Yulin	*Cremastra appendix* (D. Don) Makino	7.94%
MCG-7	Maocigu	Yunnan, Qujing	*Cremastra appendix* (D. Don) Makino	7.67%
MCG-8	Maocigu	Yunnan, Qujing	*Cremastra appendix* (D. Don) Makino	10.54%
MCG-9	Maocigu	Yunnan, Qujing	*Cremastra appendix* (D. Don) Makino	11.17%
MCG-10	Maocigu	Sichuan, Mabianshan	*Cremastra appendix* (D. Don) Makino	11.07%
MCG-11	Maocigu	Sichuan, Mabianshan	*Cremastra appendix* (D. Don) Makino	9.31%
MCG-12	Maocigu	Sichuan, Mabianshan	*Cremastra appendix* (D. Don) Makino	12.49%
BQZ-1	Bingqiuzi	Yunnan, Qujing	*Pleione bulbocodioides* (Franch.) Rolfe	10.94%
BQZ-2	Bingqiuzi	Yunnan, Qujing	*Pleione bulbocodioides* (Franch.) Rolfe	13.25%
BQZ-3	Bingqiuzi	Yunnan, Qujing	*Pleione bulbocodioides* (Franch.) Rolfe	13.68%
BQZ-4	Bingqiuzi	Guizhou, Bijie	*Pleione bulbocodioides* (Franch.) Rolfe	13.83%
BQZ-5	Bingqiuzi	Sichuan, Leshan	*Pleione bulbocodioides* (Franch.) Rolfe	13.58%
BQZ-6	Bingqiuzi	Sichuan, Leshan	*Pleione bulbocodioides* (Franch.) Rolfe	17.17%
BQZ-7	Bingqiuzi	Sichuan, Leshan	*Pleione bulbocodioides* (Franch.) Rolfe	15.16%
BQZ-8	Bingqiuzi	Yunnan, Lijiang	*Pleione bulbocodioides* (Franch.) Rolfe	13.52%
BQZ-9	Bingqiuzi	Yunnan, Lijiang	*Pleione bulbocodioides* (Franch.) Rolfe	15.71%
BQZ-10	Bingqiuzi	Yunnan, Lijiang	*Pleione bulbocodioides* (Franch.) Rolfe	15.77%
BQZ-11	Bingqiuzi	Guizhou, Qianxi	*Pleione yunnanensis* Rolfe	17.03%
BQZ-12	Bingqiuzi	Guizhou, Qianxi	*Pleione yunnanensis* Rolfe	17.42%
BQZ-13	Bingqiuzi	Guizhou, Qianxi	*Pleione yunnanensis* Rolfe	18.67%
BQZ-14	Bingqiuzi	Guizhou, Qianxi	*Pleione yunnanensis* Rolfe	18.40%

Table S2. Results of UHPLC-MS fingerprint of precision, repeatability,and stability (*n*=6).

Peak No.	Precision（RSD%）		Repeatability（RSD%）		Stability（RSD%）	
	RPA	RRT	RPA	RRT	RPA	RRT
1	2.758	0.422	2.720	0.750	2.114	0.448
2	2.756	0.734	2.484	0.631	1.804	0.840
3	2.786	0.629	1.193	0.558	1.608	0.610
4	2.808	0.384	1.161	0.781	2.271	0.713
5	2.692	0.494	1.520	0.803	2.811	0.876
6	2.512	0.197	2.551	0.449	2.139	0.472
7	2.882	0.543	2.499	0.258	2.718	0.453
8	2.969	0.100	1.735	0.155	2.808	0.319
9	1.393	0.496	2.877	0.295	2.905	0.435
10	1.480	0.265	2.446	0.104	2.706	0.410
11	2.482	0.145	2.425	0.105	2.869	0.248
12	2.442	0.164	1.182	0.094	2.947	0.174
13	2.210	0.275	0.971	0.090	2.667	0.264
14	1.586	0.164	1.999	0.125	2.702	0.260
15	2.875	0.094	2.367	0.080	2.338	0.206
16	2.319	0.078	2.796	0.067	2.028	0.168
17	2.812	0.095	2.240	0.035	2.565	0.132
18	2.514	0.054	2.585	0.071	2.433	0.154
19	1.399	0.078	2.589	0.074	2.770	0.184
20	2.850	0.079	1.373	0.090	2.186	0.155
21	2.922	0.061	2.136	0.063	1.918	0.149
22	2.394	0.023	2.528	0.060	1.968	0.168
23	2.729	0.095	2.094	0.164	2.768	0.123
24	2.541	0.148	2.819	0.168	1.641	0.139
25	2.415	0.139	2.863	0.107	2.830	0.116
26	2.351	0.076	2.730	0.099	1.439	0.060
27	2.781	0.123	2.095	0.058	0.796	0.155
28	2.013	0.181	2.965	0.048	1.210	0.152
29	2.862	0.212	2.767	0.105	1.616	0.127
30	2.559	0.048	1.725	0.081	1.858	0.025
31	2.517	0.047	2.177	0.219	1.524	0.699
32	2.905	0.037	2.680	0.058	1.922	0.275
33	1.143	0.035	2.131	0.039	1.031	0.055
34	2.831	0.034	2.198	0.061	2.434	0.076
35	2.211	0.039	2.636	0.040	2.888	0.080
36	2.087	0.075	2.203	0.053	2.537	0.072
37	1.434	0.103	1.957	0.098	2.106	0.125
38	1.796	0.116	2.011	0.123	1.994	0.163
39	2.113	0.093	2.323	0.117	1.936	0.136
40	1.392	0.068	1.798	0.092	1.773	0.098
41	1.765	0.091	2.013	0.104	2.106	0.085
42	2.004	0.057	1.826	0.073	1.850	0.088
43	1.673	0.112	1.749	0.132	2.212	0.147

Table S3. Results of similarity analysis of 12 MCG samples and 14 BQZ samples.

NO.	Similarity analysis	NO.	Similarity analysis
MCG-1	0.942	BQZ-2	0.926
MCG-2	0.945	BQZ-3	0.929
MCG-3	0.938	BQZ-4	0.922
MCG-4	0.925	BQZ-5	0.914
MCG-5	0.927	BQZ-6	0.907
MCG-6	0.931	BQZ-7	0.919
MCG-7	0.936	BQZ-8	0.920
MCG-8	0.933	BQZ-9	0.926
MCG-9	0.929	BQZ-10	0.923
MCG-10	0.941	BQZ-11	0.903
MCG-11	0.937	BQZ-12	0.886
MCG-12	0.926	BQZ-13	0.868
BQZ-1	0.922	BQZ-14	0.907

Table S4 Results of proliferative vitality efficacy indexes determination of 10 batches of MCG and 10 batches of BQZ.

Group	proliferative vitality		migration vitality	
	A549	H1299	A549	H1299
MCG-1	46.24±4.59***	59.75±3.77***	20.19±2.91***	58.19±2.91***
MCG-4	66.04±5.73***	69.51±2.34***	26.09±4.21***	70.09±4.21***
MCG-5	72.61±5.62***	79.07±4.29***	21.84±2.00***	77.84±2.00***
MCG-6	62.05±4.35***	67.45±4.90***	22.27±2.60***	68.27±2.60***
MCG-7	73.55±4.84***	64.80±4.72***	24.62±3.18***	56.62±3.18***
MCG-8	75.01±4.92***	74.53±3.80***	23.78±2.67***	68.78±2.67***
MCG-9	39.16±2.72***	43.16±3.47***	16.42±2.31***	66.42±2.31***
MCG-10	59.22±4.07***	50.86±4.87***	25.69±2.51***	67.69±2.51***
MCG-11	61.51±3.79***	57.96±4.98***	18.62±1.78***	63.62±1.78***
MCG-12	52.43±4.44***	54.92±4.68***	20.81±2.56***	58.81±2.56***
BQZ-1	60.36±2.59***	28.07±1.68***	30.28±2.62***	50.28±2.62***
BQZ-2	80.04±4.66***	42.84±7.50***	18.86±2.80***	57.86±2.80***
BQZ-3	38.85±2.12***	23.67±2.03***	14.07±2.92***	56.07±2.92***
BQZ-4	60.87±3.48***	31.91±2.89***	24.87±2.30***	60.87±2.30***
BQZ-5	33.78±3.68***	35.93±5.45***	20.33±3.26***	41.33±3.26***
BQZ-6	59.15±3.71***	46.17±1.86***	19.82±2.76***	56.82±2.76***
BQZ-7	55.08±1.55***	54.13±1.39***	15.55±1.40***	50.55±1.40***
BQZ-8	71.33±2.90***	25.70±4.45***	11.48±2.03***	65.48±2.03***
BQZ-9	66.46±6.10***	36.20±3.87***	17.24±2.11***	72.24±2.11***
BQZ-10	61.05±3.99***	56.60±2.40***	20.00±2.78***	63.00±2.78***

Table S5. Correlations and grade of GRA analysis of MCG.

Order	A549 proliferation inhibition		A549 migration inhibition		H1299 proliferation inhibition		H1299 migration inhibition	
	Peak	Correlation coefficient	Peak	Correlation coefficient	Peak	Correlation coefficient	Peak	Correlation coefficient
1	P34	0.871	P12	0.884	P15	0.869	P34	0.893
2	P6	0.863	P42	0.881	P6	0.862	P12	0.892
3	P12	0.861	P34	0.877	P34	0.859	P21	0.89
4	P15	0.842	P40	0.872	P35	0.854	P43	0.88
5	P4	0.841	P35	0.87	P26	0.851	P31	0.88
6	P21	0.84	P31	0.869	P22	0.849	P4	0.878
7	P35	0.839	P41	0.864	P17	0.846	P42	0.871
8	P20	0.836	P21	0.863	P12	0.846	P39	0.866
9	P17	0.833	P4	0.855	P39	0.843	P8	0.86
10	P24	0.833	P43	0.854	P25	0.843	P3	0.857
11	P41	0.824	P24	0.849	P20	0.841	P26	0.84
12	P42	0.823	P3	0.848	P21	0.837	P6	0.839
13	P1	0.821	P39	0.847	P24	0.836	P7	0.836
14	P31	0.818	P17	0.846	P4	0.836	P37	0.833
15	P25	0.817	P15	0.845	P7	0.833	P40	0.826
16	P39	0.816	P1	0.838	P41	0.816	P24	0.825
17	P10	0.816	P38	0.836	P31	0.815	P1	0.824
18	P43	0.814	P25	0.826	P9	0.813	P17	0.818
19	P37	0.81	P6	0.821	P13	0.811	P13	0.816
20	P19	0.809	P9	0.815	P42	0.81	P38	0.816
21	P7	0.807	P37	0.813	P19	0.807	P36	0.813
22	P8	0.807	P14	0.812	P1	0.803	P14	0.813
23	P26	0.805	P2	0.811	P43	0.802	P9	0.812
24	P40	0.8	P22	0.809	P33	0.788	P41	0.811
25	P33	0.797	P7	0.803	P40	0.788	P15	0.799
26	P9	0.796	P10	0.8	P36	0.777	P25	0.797

27	P36	0.791	P36	0.793	P10	0.777	P35	0.794
28	P3	0.79	P26	0.792	P37	0.776	P5	0.792
29	P5	0.782	P5	0.788	P3	0.766	P10	0.784
30	P13	0.777	P8	0.765	P8	0.764	P22	0.778
31	P2	0.772	P33	0.762	P38	0.76	P32	0.776
32	P22	0.771	P13	0.757	P23	0.759	P2	0.776
33	P38	0.77	P20	0.75	P5	0.745	P33	0.764
34	P14	0.753	P32	0.748	P14	0.738	P20	0.763
35	P32	0.725	P19	0.722	P2	0.735	P19	0.742
36	P23	0.715	P11	0.681	P32	0.713	P23	0.714
37	P11	0.714	P27	0.677	P30	0.668	P30	0.693
38	P30	0.713	P16	0.662	P28	0.65	P16	0.68
39	P28	0.647	P30	0.661	P11	0.649	P27	0.659
40	P27	0.646	P23	0.652	P27	0.627	P28	0.657
41	P16	0.627	P28	0.639	P16	0.623	P11	0.65
42	P18	0.605	P29	0.605	P29	0.592	P29	0.594
43	P29	0.592	P18	0.603	P18	0.576	P18	0.583

Table S6. Correlations and grade of GRA analysis of BQZ.

Order	A549 proliferation inhibition		A549 migration inhibition		H1299 proliferation inhibition		H1299 migration inhibition	
	Peak	Correlation coefficient	Peak	Correlation coefficient	Peak	Correlation coefficient	Peak	Correlation coefficient
1	P5	0.877	P35	0.929	P6	0.887	P38	0.93
2	P34	0.868	P39	0.911	P34	0.885	P35	0.919
3	P24	0.866	P38	0.905	P24	0.874	P43	0.913
4	P6	0.864	P34	0.902	P10	0.865	P28	0.912
5	P35	0.863	P18	0.894	P14	0.865	P39	0.912
6	P38	0.861	P43	0.892	P17	0.863	P34	0.91
7	P39	0.857	P24	0.891	P43	0.863	P32	0.906
8	P43	0.857	P12	0.888	P12	0.862	P4	0.898
9	P12	0.851	P32	0.888	P38	0.862	P31	0.897
10	P41	0.841	P31	0.887	P37	0.861	P10	0.89
11	P17	0.841	P10	0.875	P5	0.86	P33	0.889
12	P18	0.84	P41	0.869	P33	0.86	P40	0.887
13	P31	0.838	P14	0.867	P39	0.859	P41	0.886
14	P42	0.838	P13	0.866	P4	0.857	P9	0.876
15	P15	0.837	P42	0.865	P18	0.855	P37	0.874
16	P20	0.837	P6	0.865	P20	0.853	P6	0.872
17	P14	0.835	P9	0.864	P7	0.849	P2	0.87
18	P37	0.834	P40	0.864	P32	0.844	P12	0.867
19	P10	0.834	P33	0.863	P31	0.843	P18	0.859
20	P8	0.833	P7	0.861	P37	0.838	P24	0.856
21	P9	0.832	P2	0.858	P27	0.838	P42	0.852
22	P32	0.832	P8	0.847	P9	0.835	P17	0.852
23	P4	0.83	P11	0.843	P30	0.834	P7	0.85
24	P33	0.827	P17	0.843	P40	0.834	P5	0.849
25	P25	0.823	P21	0.841	P13	0.832	P21	0.848
26	P16	0.822	P25	0.84	P21	0.832	P8	0.847

27	P40	0.818	P5	0.836	P16	0.831	P20	0.842
28	P11	0.811	P20	0.836	P15	0.829	P14	0.839
29	P2	0.809	P37	0.831	P2	0.828	P16	0.839
30	P27	0.808	P16	0.83	P7	0.828	P15	0.838
31	P30	0.806	P4	0.825	P41	0.824	P25	0.838
32	P7	0.804	P30	0.822	P42	0.82	P11	0.828
33	P22	0.801	P27	0.806	P22	0.817	P30	0.828
34	P3	0.801	P15	0.801	P11	0.816	P3	0.816
35	P21	0.795	P3	0.795	P25	081	P27	0.8
36	P28	0.788	P19	0.794	P3	0.81	P19	0.788
37	P19	0.785	P23	0.765	P19	0.805	P22	0.787
38	P23	0.777	P22	0.76	P28	0.801	P1	0.776
39	P13	0.773	P1	0.756	P23	0.8	P23	0774
40	P1	0.752	P29	0.744	P1	0.781	P31	0.765
41	P26	0.744	P28	0.74	P26	0.745	P29	0.753
42	P29	0.696	P26	0.709	P29	0.735	P26	0.724
43	P36	0.638	P36	0.603	P36	0.618	P36	0.629

Table S7. Correlations and grade of BCA analysis of MCG.

Order	A549 proliferation inhibition		A549 migration inhibition		H1299 proliferation inhibition		H1299 migration inhibition	
	Peak	Correlation coefficient	Peak	Correlation coefficient	Peak	Correlation coefficient	Peak	Correlation coefficient
1	P34	0.789	P25	0.533	P22	0.809	P42	0.624
2	P6	0.783	P42	0.493	P34	0.789	P6	0.591
3	P12	0.671	P19	0.486	P25	0.717	P34	0.546
4	P35	0.626	P41	0.475	P6	0.714	P26	0.543
5	P15	0.579	P40	0.473	P26	0.705	P1	0.538
6	P19	0.577	P15	0.461	P23	0.691	P21	0.535
7	P22	0.568	P34	0.457	P15	0.675	P12	0.519
8	P42	0.556	P6	0.43	P19	0.635	P22	0.489
9	P20	0.551	P35	0.416	P20	0.595	P23	0.444
10	P1	0.545	P1	0.384	P12	0.572	P2	0.31
11	P25	0.523	P20	0.364	P4	0.567	P25	0.252
12	P4	0.471	P21	0.336	P35	0.54	P19	0.203
13	P24	0.415	P12	0.31	P39	0.536	P20	0.173
14	P26	0.403	P2	0.301	P24	0.474	P28	0.154
15	P41	0.398	P4	0.3	P13	0.43	P39	0.118
16	P23	0.349	P22	0.295	P17	0.415	P8	0.106
17	P21	0.317	P23	0.215	P21	0.343	P24	0.067
18	P2	0.247	P17	0.205	P41	0.338	P17	0.056
19	P17	0.232	P31	0.156	P1	0.325	P3	0.05
20	P10	0.231	P26	0.143	P9	0.172	P15	0.049
21	P11	0.189	P13	0.142	P42	0.162	P7	0.039
22	P37	0.15	P38	0.142	P7	0.125	P4	0.037
23	P39	0.14	P24	0.141	P43	0.079	P10	0.033
24	P40	0.092	P14	0.122	P2	0.012	P36	-0.02
25	P13	0.087	P10	0.119	P28	-0.004	P37	-0.034
26	P8	0.049	P37	0.06	P8	-0.044	P11	-0.072

27	P9	0.038	P28	-0.063	P10	-0.096	P43	-0.088
28	P7	0.013	P11	-0.069	P37	-0.102	P35	-0.114
29	P3	0.01	P30	-0.111	P31	-0.129	P13	-0.116
30	P31	-0.002	P14	-0.122	P40	-0.15	P16	-0.18
31	P43	-0.025	P5	-0.126	P33	-0.251	P9	-0.192
32	P14	-0.055	P8	-0.162	P30	-0.255	P29	-0.256
33	P33	-0.178	P43	-0.167	P11	-0.274	P41	-0.281
34	P28	-0.209	P39	-0.194	P36	-0.289	P14	-0.282
35	P5	-0.213	P33	-0.235	P3	-0.313	P30	-0.288
36	P36	-0.273	P27	-0.252	P5	-0.331	P32	-0.388
37	P30	-0.289	P29	-0.314	P16	-0.454	P18	-0.566
38	P18	-0.292	P18	-0.317	P14	-0.469	P27	-0.579
39	P38	-0.341	P7	-0.343	P38	-0.496	P31	-0.602
40	P27	-0.358	P16	-0.353	P18	-0.522	P33	-0.636
41	P16	-0.365	P36	-0.394	P32	-0.58	P5	-0.661
42	P29	-0.515	P9	-0.468	P27	-0.6	P40	-0.803
43	P32	-0.588	P32	-0.665	P29	-0.619	P38	-0.886

Table S8. Correlations and grade of BCA analysis of BQZ.

Order	A549 proliferation inhibition		A549 migration inhibition		H1299 proliferation inhibition		H1299 migration inhibition	
	Peak	Correlation coefficient	Peak	Correlation coefficient	Peak	Correlation coefficient	Peak	Correlation coefficient
1	P24	0.535	P35	0.755	P24	0.671	P38	0.469
2	P5	0.51	P42	0.548	P38	0.551	P37	0.322
3	P35	0.507	P24	0.501	P6	0.513	P3	0.314
4	P38	0.442	P12	0.469	P20	0.497	P43	0.291
5	P39	0.427	P38	0.461	P4	0.482	P15	0.228
6	P42	0.404	P39	0.449	P34	0.469	P2	0.193
7	P34	0.387	P1	0.411	P28	0.466	P4	0.127
8	P20	0.355	P20	0.358	P5	0.454	P10	0.114
9	P6	0.351	P34	0.348	P12	0.401	P28	0.113
10	P4	0.337	P18	0.334	P27	0.392	P30	0.098
11	P28	0.308	P11	0.305	P30	0.359	P33	0.096
12	P12	0.306	P30	0.267	P18	0.278	P22	0.08
13	P27	0.235	P4	0.242	P19	0.223	P1	0.07
14	P41	0.198	P43	0.239	P14	0.222	P5	0.056
15	P25	0.186	P5	0.218	P23	0.215	P24	0.051
16	P17	0.164	P25	0.205	P43	0.195	P12	0.05
17	P22	0.156	P2	0.196	P39	0.194	P6	0.05
18	P14	0.124	P7	0.151	P1	0.19	P39	0.047
19	P18	0.081	P27	0.15	P22	0.188	P16	0.04
20	P30	0.071	P21	0.148	P35	0.156	P27	0.033
21	P36	0.066	P6	0.144	P21	0.149	P35	0.027
22	P1	0.044	P28	0.129	P42	0.143	P23	0.004
23	P23	0.038	P3	0.114	P33	0.142	P25	0.003
24	P43	0.022	P16	0.026	P3	0.133	P11	-0.014
25	P11	-0.006	P32	-0.012	P8	0.125	P20	-0.038
26	P19	-0.045	P21	-0.016	P17	0.118	P19	-0.046

27	P8	-0.048	P23	-0.038	P15	0.112	P42	-0.06
28	P3	-0.094	P41	-0.04	P16	0.098	P41	-0.068
29	P16	-0.134	P8	-0.043	P2	0.074	P34	-0.071
30	P37	-0.151	P40	-0.052	P11	0.054	P18	-0.142
31	P2	-0.186	P10	-0.062	P7	-0.008	P8	-0.144
32	P21	-0.193	P36	-0.084	P37	-0.035	P14	-0.165
33	P15	-0.201	P19	-0.092	P25	-0.043	P23	-0.213
34	P26	-0.202	P17	-0.1	P10	-0.056	P7	-0.219
35	P9	-0.217	P14	-0.1	P41	-0.228	P36	-0.239
36	P10	-0.254	P26	-0.129	P36	-0.342	P13	-0.327
37	P40	-0.283	P31	-0.143	P26	-0.406	P17	-0.357
38	P7	-0.3	P29	-0.143	P9	-0.483	P32	-0.632
39	P33	-0.303	P33	-0.156	P40	-0.538	P26	-0.634
40	P13	-0.306	P15	-0.185	P15	-0.545	P9	-0.643
41	P32	-0.588	P9	-0.249	P32	-0.616	P31	-0.703
42	P31	-0.634	P37	-0.271	P29	-0.717	P29	-0.703
43	P29	-0.634	P13	-0.382	P31	-0.717	P40	-0.769

Table S9. The content and proportion of each monomer in MCGC.

Monomer component	Content of crude MCG-9 (μg·g^{-1})	Proportion (%)
monbarbatain A	12.47	0.039
blestriarene A	24.47	0.077
blestriarene B	33.91	0.107
2,7-dihydroxy-1-(4-hydroxybenzyl)-4-methoxyphenanthrene	7.11	0.022
coelonin	100.72	0.317
batatasin III	111.94	0.353
2-(*p*-hydroxybenzyl)-3',5-dihydroxy-3-methoxybibenzyl	308.96	0.974
gastrodin	14119.43	44.504
2-isobutylmalic acid	4447.74	14.019
malic acid	11567.88	36.461
citric acid	991.65	3.126

Table S10. The content and proportion of each monomer in BQZC.

Monomer component	Content of crude BQZ-3 (μg·g^{-1})	Proportion (%)
monbarbatain A	4.19	0.008
blestriarene A	35.06	0.066
blestriarene B	20.56	0.039
2,7-dihydroxy-1-(4-hydroxybenzyl)-4-methoxyphenanthrene	4.56	0.009
coelonin	128.8	0.244
batatasin III	197.03	0.373
2-(*p*-hydroxybenzyl)-3',5-dihydroxy-3-methoxybibenzyl	294.81	0.558
gastrodin	22713.24	42.997
2-isobutylmalic acid	5569.23	10.543
malic acid	20676.03	39.14
citric acid	3181.66	6.023

Table S11. Comparison of tumor inhibition rates among different groups.

**Groups**	**Inbition rates of lung cancer（%）**
Y	78.19
MCG-9	56.83
MCGC	58.30
BQZ-3	58.96
BQZC	60.84
